# Targeting inhibitory cerebellar circuitry to alleviate behavioral deficits in a mouse model for studying idiopathic autism

**DOI:** 10.1038/s41386-020-0656-5

**Published:** 2020-03-16

**Authors:** Owen Y. Chao, Ezequiel Marron Fernandez de Velasco, Salil Saurav Pathak, Swati Maitra, Hao Zhang, Lisa Duvick, Kevin Wickman, Harry T. Orr, Hirokazu Hirai, Yi-Mei Yang

**Affiliations:** 1 Department of Biomedical Sciences, University of Minnesota Medical School, Duluth, MN 55812 USA; 2 Department of Pharmacology, University of Minnesota, Minneapolis, MN 55455 USA; 3 Institute for Translational Neuroscience, University of Minnesota, Minneapolis, MN 55455 USA; 4 Department of Laboratory Medicine and Pathology, University of Minnesota, Minneapolis, MN 55455 USA; 5 Department of Neurophysiology and Neural Repair, Gunma University Graduate School of Medicine, Maebashi, Gunma, 371-8511 Japan; 6 Department of Neuroscience, University of Minnesota, Minneapolis, MN 55455 USA

**Keywords:** Excitability, Autism spectrum disorders

## Abstract

Autism spectrum disorder (ASD) encompasses wide-ranging neuropsychiatric symptoms with unclear etiology. Although the cerebellum is a key region implicated in ASD, it remains elusive how the cerebellar circuitry is altered and whether the cerebellum can serve as a therapeutic target to rectify the phenotype of idiopathic ASD with polygenic abnormalities. Using a syndromic ASD model, e.g., Black and Tan BRachyury T^+^Itpr3^tf^/J (BTBR) mice, we revealed that increased excitability of presynaptic interneurons (INs) and decreased intrinsic excitability of postsynaptic Purkinje neurons (PNs) resulted in low PN firing rates in the cerebellum. Knowing that downregulation of Kv1.2 potassium channel in the IN nerve terminals likely augmented their excitability and GABA release, we applied a positive Kv1.2 modulator to mitigate the presynaptic over-inhibition and social impairment of BTBR mice. Selective restoration of the PN activity by a new chemogenetic approach alleviated core ASD-like behaviors of the BTBR strain. These findings highlight complex mechanisms converging onto the cerebellar dysfunction in the phenotypic model and provide effective strategies for potential therapies of ASD.

## Introduction

Autism spectrum disorder (ASD) is a neurodevelopmental disorder with an incidence of 1 in 59 children, characterized by defective social interaction, impaired communication and restricted patterns of repetitive behaviors [1, 2]. The “spectrum” reflects vast heterogeneity in its causes and symptoms. Human genetic analyses identify a multitude of genes associated with ASD [3–5]. Yet, many of them are not restricted to ASD but rather overlap with other neuropsychiatric disorders [6, 7]. The occurrence for the majority of ASD is reported as idiopathic. To understand the mechanisms of ASD, tremendous progress is made by generating animal models that mimic genetic disorders related to ASD. For instance, knockout (KO) mice of *Fmr1*, *Mecp2*, *Shank3*, and *Tsc1/2* model Fragile X syndrome (FXS), Rett syndrome, Phelan-McDermid syndrome, and Tuberous sclerosis complex, respectively [8–12].

Consistent with the heterogeneous causes, symptoms of ASD are highly diverse with individual severity. Besides the key characters, cognitive and psychiatric deficits are frequently present in ASD [1, 2]. Among several mouse lines that simulate the multifaceted traits of ASD, Black and Tan BRachyury T^+^Itpr3^tf^/J (BTBR) mice are widely adopted [13, 14]. They display reduced sociability, altered ultrasonic vocalizations during development and increased self-grooming behavior. We have validated that they also exhibit the psychiatric comorbidity of ASD, including anxiety and attention deficit [15]. Additionally, these mice share similarities of anatomical changes with ASD patients [16], strengthening their applicability as an animal model for studying idiopathic autism [17].

Given the etiological and phenotypical complexity of ASD, a direct approach is to investigate the brain regions that are evidently modified in the condition. Although ASD involves many brain areas, mounting evidence suggests the cerebellum is highly engaged in the pathogenesis of ASD [18–20]. Notwithstanding variability in clinical manifestations, abnormal, typically reduced, number of Purkinje neurons (PNs) [21], and cerebellar vermal volume [22] (but see ref. [23]), decreased cerebellum activation during cognitive and social processing [24, 25] and disrupted cerebro-cerebellar functional connectivity [26] are linked to ASD. PN-specific deletion of ASD-related genes in mice impairs the intrinsic excitability of PNs and/or their synaptic responses, resulting in autistic behaviors [27–30]. Compared to the single-gene mutations [8–12, 27–30], the cellular and molecular underpinnings of the BTBR strain with polygenic abnormalities are not well defined. As the model may present translational values for the search for therapeutics for ASD of an idiopathic nature, it is valuable to address how the cerebellar circuitry is affected and whether targeting the cerebellum activity is operative to rescue the phenotypes of BTBR mice.

By patch-clamp recordings, we find that PNs fire at low frequencies in the BTBR cerebellum as compared to C57BL/6J (wild type, WT) mice. After blocking excitatory and/or inhibitory synaptic inputs, we reveal that the reduced PN firing is due to enhanced GABA release from interneurons (INs) and lessened intrinsic excitability of PNs. We identify Kv1.2, a low-threshold K^+^ channel expressed in the axonal terminal of INs, is downregulated, which likely accounts for the increased presynaptic excitability and GABA release at the BTBR synapses. Application of a Kv1.2 agonist, docosahexaenoic acid (DHA), alleviates the inhibitory overtone in vitro and social deficits of BTBR animals in vivo. Selectively boosting the membrane excitability of PNs with a cell-type specific chemogenetic method restores the PN spontaneous activity and rectifies the autistic-like behaviors of the idiopathic ASD model.

## Materials and methods

Experimental details are in the supplementary Materials and Methods section.

### Subjects

BTBR and C57BL/6J mice were from Jackson Laboratory and housed in a facility accredited by the Association for the Assessment and Accreditation of Laboratory Animal Care. All procedures were approved by the Institutional Animal Care and Use Committee and the Institutional Biosafety Committee of University of Minnesota, in accordance with the National Institutes of Health guidelines. Male mice were used unless otherwise specified. Choice of sexes was based on the male-dominant prevalence of ASD [2]. Different batches of animals were used for each set of experiments.

### Electrophysiology

28–35-days-old mice were subject to electrophysiological and other analyses (except for behavioral testing). Following decapitation, the brain was dissected and sagittal cerebellar slices were sectioned at a thickness of 300 µm in ice-cold modified artificial cerebral spinal fluid (ACSF). It contained (in mM): sucrose (217.6), KCl (3), glucose (10), NaH_2_PO_4_ (2.5), NaHCO_3_ (26), MgCl_2_ (2), and CaCl_2_ (2), continuously bubbled in 95% O_2_ and 5% CO_2_ (pH 7.4). Subsequently, slices were incubated in oxygenated standard ACSF including (in mM): NaCl (125), KCl (2.5), glucose (10), NaH_2_PO_4_ (1.25), sodium pyruvate (2), myo-inositol (3), ascorbic acid (0.5), NaHCO_3_ (26), MgCl_2_ (1), and CaCl_2_ (2) (pH 7.4) at 37 °C for 30 min.

Slices were perfused with the standard ACSF with supplement of NBQX (10 µM) and APV (50 µM) to block AMPA and NMDA receptors or bicuculline (10 µM) to block GABA_A_ receptors. Patch electrodes had resistances of 2.5–3 and 4.5–6 MΩ for PNs and INs, respectively. To record EPSCs, the intracellular solution contained (in mM): K-gluconate (97.5), CsCl (32.5), EGTA (5), HEPES (10), MgCl_2_ (1), TEA (30), and lidocaine *N-*ethyl bromide (3) (pH 7.3). To record IPSCs, the intracellular solution contained (in mM): K-gluconate (50), CsCl (80), EGTA (5), HEPES (10), MgCl_2_ (1), TEA (30) and lidocaine N-ethyl bromide (3) (pH 7.3). APs were recorded in the cell-attached mode with GΩ seal at −60 mV for PNs and −70 mV for INs. The intracellular solution included (in mM): K-gluconate (97.5), KCl (32.5), EGTA (0.1), HEPES (40), MgCl_2_ (1), ATP (2), GTP (0.5) (pH 7.3). The same solution was used to record spikes from PNs in the current-clamp mode at −70 mV.

All recordings were acquired on-line at ~23 °C, filtered at 4 kHz, digitized at 50 kHz with an amplifier (MultiClamp 700B) and digitizer (Digidata 1550B). Data were analyzed off-line with MiniAnalysis 6.0.7 (Synaptosoft), Clampfit 10 (Molecular Devices) and Excel 2016 (Microsoft). Reagents were from Millipore Sigma, Tocris Bioscience and Alomone Labs.

### Immunohistochemistry

Mice were anesthetized, and perfused with ice-cold phosphate-buffered saline (PBS, pH 7.4), followed by 4% paraformaldehyde. Brains were extracted, immersed in 4% paraformaldehyde at 4 °C overnight, and transferred to 30% sucrose solution at 4 °C until they sank. They were sectioned into 50 µm-thick slices, maintained in 0.3% H_2_O_2_ solution and rinsed in PBS. Brain slices were permeabilized with 0.2% Triton X-100 and labeled with primary antibodies for 48 h at 4 °C. They were incubated in biotinylated anti-mouse antibody (1:200; BA-9200) for 2 h and then in ABC reagent (PK-4000) for 1 h. Slices were stained with 3,3′-diaminobenzidine (DAB; SK-4100) and washed with distilled water, mounted (H-5000) and cover-slipped. To add fluorescence, after incubation in primary antibodies, slices were incubated in secondary antibodies (1:1000; Invitrogen Alex Fluor 488, 555, or 647) for 3 h and then mounted and cover-slipped with mounting medium (P36966).

Primary antibodies included: anti-Kv1.2 (1:1000; SAB5200059) and anti-Calbindin-D28K (1:1000; PA1–931), and Parvalbumin (1:1000; NBP2-50036).

### Western blotting

The cerebellum of BTBR and WT mice were dissected and snap-frozen in dry ice. Brain tissues were homogenized in ice-cold lysis buffer (20 mM HEPES pH7.5, 100 mM NaCl, 0.05% Triton X-100, 1 mM DTT, 5 mM Na-Betaglycerophosphate, 0.5 mM Na-vanadate, 1 mM EDTA, protease inhibitors) and protein was extracted and quantified by Bradford assay. Fifty microgram protein lysates were resolved on a 10% SDS polyacrylamide gel and transferred to PVDF membrane. Membranes were blocked by 5% non-fat dry milk and incubated with primary antibodies overnight at 4 °C. Next day blots were washed and incubated in horseradish peroxidase (HRP) conjugated with anti-rabbit or anti-mouse IgG antibodies (1:5000; Bio-Rad) for 1.5 h and washed again. To detect immunoreactivity, blots were incubated with enhanced chemiluminescent reagent (Perkin Elmer) and imaged on Odyssey Fc Imaging System (LI-COR Biosciences). Relative intensity of blots was quantified using ImageJ-NIH software.

Primary antibodies were: anti-Kv1.2 (1:1000; SAB5200059), anti-FMRP (1:100; NBP2-01770), anti-pFMRP (1:50; ab48127), anti-mTOR (1:1000; 2983S), anti-p-mTOR (1:1000; 2971S), anti-ERK (1:1000; sc-514302), anti-p-ERK (1:1000; 9101S), anti-mGluR5 (1:100; PA1-38132), and anti-β-actin (1:5000; A5441).

### DHA administration in vivo

2–3-months-old BTBR mice underwent behavioral testing before and after intraperitoneal (i.p.) injection of cis-4,7,10,13,16,19-Docosahexaenoic acid sodium salt (DHA; D8768) or sterile saline. Tests were conducted 30 min after DHA (200 mg/kg) or saline injection to minimize distress from drug administration and ensure normal physical activity that could be affected by DHA for a short period [31].

### Adeno-associated virus (AAV)-vector delivery

AAV8-Pcp2-hM3Dq-mCherry and AAV8-Pcp2-mCherry vectors (titers > 4.0 × 10^13^) were prepared by the University of Minnesota Viral Vector and Cloning Core following standard packaging procedures. Minimal Pcp2/L7 promoter was inserted into pAAV-CaMKIIa-hM3D(Gq)-mCherry (a gift from Dr. Bryan Roth; Addgene #50476) to confer specific expression of human M3 muscarinic receptor (hM3Dq) and/or mCherry to PNs [32]. Activation of hM3Dq by clozapine-*N*-oxide (CNO, BML-NS105) increased neuronal excitability [33].

#### Intracranial injection to cover the whole cerebellum

BTBR pups at P5 were randomly assigned to AAV8-Pcp2-hM3Dq-mCherry or AAV8-Pcp2-mCherry group and anesthetized by hypothermia. The viral vectors (1:3 dilution) were slowly injected into the loci close to the cerebellum bilaterally (1.5–2.0 µl each side). At ~P30, the animals underwent behavioral tests following CNO administration (1 mg/kg, i.p.).

#### Infusion into the cerebellum subregions via stereotaxic surgery

2–3-months-old BTBR or WT mice were anaesthetized with ketamine (100 mg/kg, i.p.) and xylazine (10 mg/kg, i.p.). Mice were mounted on a stereotaxic frame and holes were drilled according to the coordinates: AP: −7.0 mm, ML: ±1.0 mm, relative to the bregma, to target lobules VI and VIIa. AAV8-Pcp2-hM3Dq-mCherry (0.2 µl each side) was infused 0.5 mm below brain surface at a 0.1 µl/min flowrate. Three weeks after surgery, behavioral tests were conducted with a within-group design.

### Behavioral testing

Behaviors were recorded via a camera connected to a tracking software ANY-maze. Experimenters were blind to the treatments. DHA, CNO or saline was given 30–40 min before each test.

#### Three-chamber social test

The test was to evaluate mouse sociable behaviors, consisting of three trials: habituation, sociability and social novelty [14]. Each trial lasted for 9 min. Physical contacts around the cups with the nose, head and forelimbs were defined as exploration behaviors. To minimize individual differences, sociable index = (time for exploring the stranger—time for exploring the empty cup) / (total exploration time); and social novelty index = (time for exploring the novel stranger—time for exploring the familiar stranger) / (total exploration time) were calculated.

#### Elevated open platform

The test exploited the innate tendency of fear of open space in rodents [15]. Animals were put onto an open platform that was elevated 30 cm for 5 min. Distance traveled and time spent in the center area were recorded.

#### Open field

The test was to measure general locomotor and exploratory activities [34]. Mice were put into the arena for 10–15 min. Distance traveled, rearing, grooming, thigmotactic behaviors, entries, and time stayed at the center were analyzed.

#### Object-based attention test

The test was to measure attention-associated processes and/or working (short-term) object memory [15]. It was identical to a novel object-preference test, except with no time delay between learning and test trials. Each trial lasted for 5 min. An index was calculated: index = (time for exploring the novel object—time for exploring the old object) / (total object exploration time).

### Statistics

Mixed two-way ANOVAs with a “between-subject” factor (group) and a “within-subject” factor (condition, object or interval), and repeated two-way ANOVAs with “within-subject” factors (treatment, condition, object and interval) were applied to analyze the data. When a significant effect was detected, one-way or repeated one-way ANOVAs and LSD post hoc tests were conducted. Paired Student’s *t*-tests and Mann–Whitney *U*-test were used when appropriate. One-sample *t*-tests were applied to compare the indexes to zero value. All statistics were two-tailed and significant levels were set as *p* < 0.05. Data were expressed as the mean ± standard error (s.e.m.). For electrophysiology, *n* denoted the number of cells from >3 animals in each group. For other analyses, *n* represented the number of mice. Sample sizes were decided by previous studies using similar protocols [15, 31].

## Results

### Reduced output from the BTBR cerebellar cortex

**Fig. 1:**
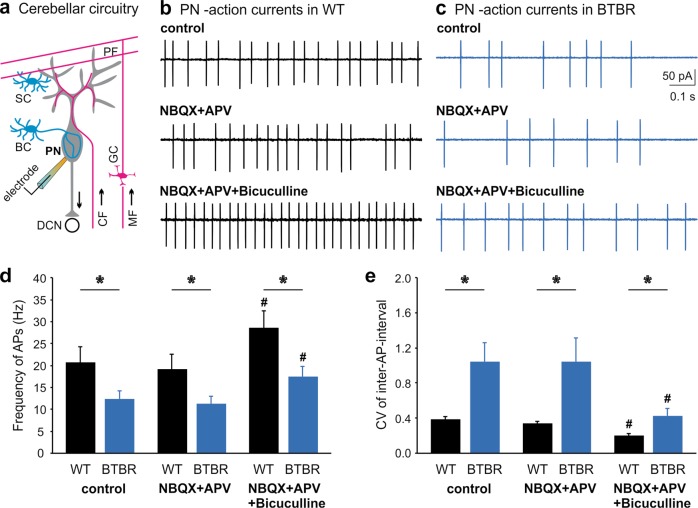
Reduced firing activity of PNs in the BTBR cerebellum. **a** Schematics of cerebellar circuitry including excitatory and inhibitory inputs to a PN. BC basket cell, SC stellate cell, DCN deep cerebellar nuclei, CF climbing fiber, MF mossy fiber, PF parallel fiber, GC granule cell. (**b**, **c** Representative action currents (i.e., APs in the voltage-clamp mode) from PNs recorded in the cell-attached configuration from WT (**b**) and BTBR (**c**) brain slices in the absence (control, top panels) or presence of AMPA-receptor and NMDA-receptor blockers NBQX (10 μM) and APV (50 μM) (middle panels), or in a combination of NBQX, APV, and a GABA_A_ receptor blocker bicuculline (10 μM, bottom panels). **d** Summary of frequency of APs from WT (black bars, *n* = 10) and BTBR (blue bars, *n* = 10) PNs in the aforementioned conditions (**b**, **c**). The frequency is the reciprocal of each inter-AP-intervals. **e** Summarized coefficient of variation (CV) of inter-AP-intervals for the same WT (black bars, *n* = 10) and BTBR (blue bars, *n* = 10) neurons. ^#^ indicates significant differences between “NBQX + APV + bicuculline” and “control” conditions within each group. Data are represented as mean ± s.e.m. Asterisks (*) or “ns” denotes statistical significance (*p* < 0.05) or “not significant” respectively in this and following figures.

In the cerebellar circuitry, PNs integrate excitatory inputs from parallel fibers (PFs) of granule cells and climbing fibers (CFs) of the inferior olivary nucleus; and inhibitory inputs from molecular layer INs, namely basket cells (BCs) and stellate cells (SCs) [35, 36]. Being the sole output from the cerebellar cortex, PNs send GABAergic projections to deep cerebellar nuclei (DCN) to control the cerebello-thalamo-cortex connectivity (Fig. 1a). We made cell-attached patch clamp recordings of spontaneous action potentials (APs) from PNs in the vermal folia V–VII of sagittal cerebellar slices taken from BTBR and their age and background-matched WT mice at postnatal days (P) 28–35. The firing frequency of PNs in BTBR dramatically reduced in comparison with WT animals (*F*_1,18_ = 4.679, *p* = 0.044; Fig. 1b–d). Eliminating excitatory inputs with NBQX (AMPA receptor antagonist) and APV (NMDA receptor antagonist) did not affect the spontaneous firing of either WT or BTBR PNs. In contrast, further inhibiting GABA_A_ receptors with bicuculline increased the firing rates of BTBR (*p* = 0.009, post-hoc) and WT (*p* = 0.001, post-hoc) neurons, compared to the control condition (Supplementary Table S1). This reinforces that inhibition but not excitation plays a direct role in determining the PN spontaneous activity. Although blocking inhibitory inputs alleviated the firing deficit, the frequency of APs from BTBR PNs remained substantially lower than that from the WT group (*F*_1,18_ = 6.171, *p* = 0.023), implying reduced intrinsic excitability of these neurons. To analyze the regularity of PN firing, we calculated the coefficient of variation (CV) of inter-spike-intervals and summarized in Fig. 1e. Across all the conditions, BTBR PNs elicited APs less regularly with poorer precision than WT ones (control: *F*_1,18_ = 9.115, *p* = 0.007; NBQX + APV: *F*_1,18_ = 6.764, *p* = 0.018; NBQX + APV + bicuculline: *F*_1,18_ = 7.745, *p* = 0.012), despite that removal of inhibitory transmission by bicuculline limited the firing variabilities for both groups. Taken together, these observations suggest that abnormal inhibition from presynaptic INs and/or excitability of postsynaptic PNs mediate the reduced output from the BTBR cerebellar cortex.

### Increased GABA release from INs and decreased intrinsic excitability of PNs account for low PN activity in the BTBR cerebellum

**Fig. 2:**
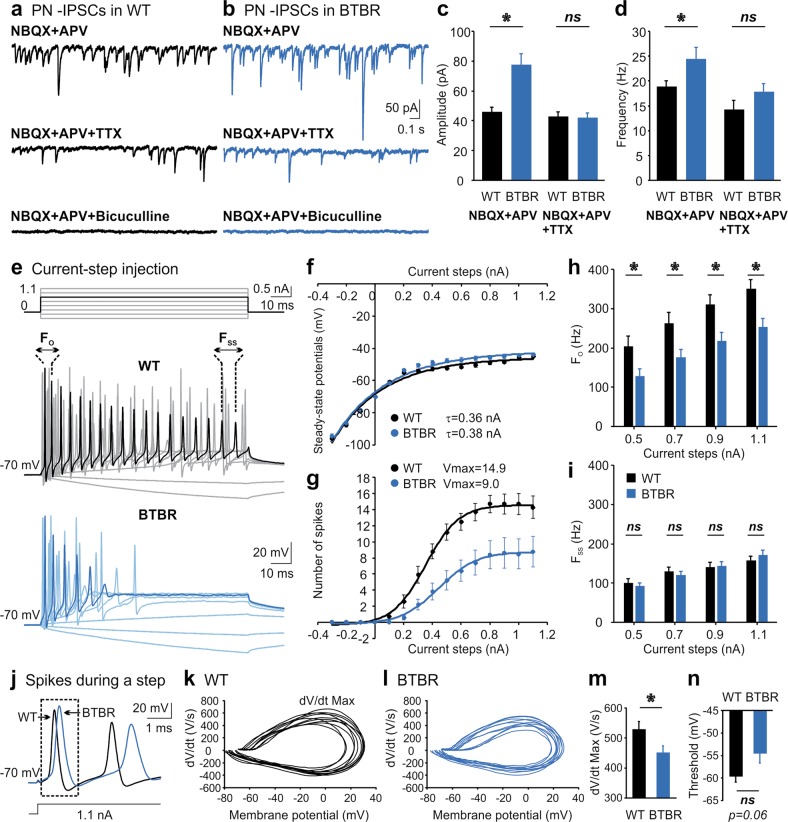
Excessive GABA release from INs and decreased intrinsic excitability of PNs in the BTBR synapses. **a**, **b** IPSCs recorded in the whole-cell mode at a holding potential of −60 mV from PNs of WT (**a**) and BTBR (**b**) mice in NBQX (10 μM) and APV (50 μM) to block excitatory inputs. The same PNs were then exposed to TTX (1 μM) or bicuculline (10 μM). **c**, **d** Summary of the amplitude (**c**) and frequency (**d**) of IPSCs for WT (black bars) and BTBR (blue bars) PNs before (*n* = 16 for WT and BTBR) and after TTX exposure (*n* = 13 for WT; *n* = 9 for BTBR). The frequency is calculated as the reciprocal of each inter-IPSC-intervals and averaged for each neuron. **e** APs evoked by current steps (top) from a WT (middle) or BTBR (bottom) PN. All synaptic inputs are blocked by NBQX (10 μM), APV (50 μM) and bicuculline (10 μM). **f**, **g** Steady-state potentials (**f**) measured within last 5 ms of each evoked potential and number of spikes (**g**) generated by the current steps in WT (*n* = 10, black) and BTBR (*n* = 10, blue) neurons. Solid lines represent fits to a single exponential function: *f*(*t*) = Ae^−t/τ^ +C (**f**) or a Boltzmann function: *f*(*I*) = Vmax/(1 + e^(Imid-I)/Ic^)+C (**g**). “Vmax” is theoretical value of the maximal number of APs; “Imid” is depolarization current needed to produce half of the maximal number of APs; “Ic” is steepness of the Boltzmann curve. **h**, **i** Summary of F_O_ (**h**) and F_SS_ (**i**) for WT (*n* = 10, black) and BTBR (*n* = 10, blue) PNs. F_O_ and F_SS_ are derived from the first and last inter-spike intervals, respectively (**e**). (**j**) Overlay of first spikes from a WT (black) and a BTBR (blue) PN, evoked by a current step of 1.1 nA. **k**, **l** Phase plane plots of the first spikes from WT (**k**) and BTBR (**l**) cells. **m**, **n** Maximal value of d*V*/d*t* (d*V*/d*t* Max, **m**) and spike threshold, i.e., the voltage where d*V*/d*t* reaches 5% of its maximum (**n**) are summarized for WT (*n* = 10, black bars) and BTBR (*n* = 10, blue bars) groups.

To directly investigate synaptic contributions to PN firing, we recorded spontaneous inhibitory postsynaptic currents (sIPSCs) by whole-cell voltage clamping PNs at a holding potential of −60 mV in the presence of NBQX and APV (Fig. 2a, b). sIPSCs from BTBR PNs were significantly larger in the amplitude (*F*_1,30_ = 16.75, *p* < 0.001) and more frequent (*F*_1,30_ = 4.926, *p* = 0.034) than those from WT cells (Fig. 2c, d). Inhibiting presynaptic APs with tetrodotoxin (TTX, a Na^+^ channel blocker) to obtain miniature IPSCs (mIPSCs) abolished the differences between the two groups (*p* > 0.05). Addition of bicuculline wiped out all the events, confirming their GABAergic origin. These results implicate that presynaptic quantal release and postsynaptic GABA_A_ receptors mediating mIPSCs are intact in the BTBR cerebellum. The augmented sIPSCs are likely driven by AP-dependent GABA release from INs.

On the contrary, miniature or spontaneous excitatory postsynaptic currents (mEPSCs or sEPSCs), measured in bicuculline with or without TTX, did not show drastic changes, except for an increased frequency of sEPSCs in the BTBR neurons (*F*_1,21_ = 5.544, *p* = 0.028; Supplementary Fig. S1). As PFs and CFs respectively innervate the distal and proximal dendritic spines of PNs, such an increase in the small and spare synaptic currents unlikely generate sufficient depolarizing conductance to facilitate the firing of large and “leaky” PNs [37]. This is also evident in Fig. 1 where elimination of excitatory inputs had no effect on the PN firing.

Next, we examined the intrinsic excitability of PNs in the whole-cell current clamp mode after blocking excitatory and inhibitory neurotransmission. A series of current steps were delivered to PNs at the membrane potential of −70 mV, where spontaneous spiking was suppressed (Fig. 2e). The repetitive spikes were responses only to the supra-threshold current injections. By measuring the steady-state potential produced by each current step, the current–voltage (I–V) relationships for WT and BTBR PNs virtually overlapped, indicating the input resistance of PNs was the same (Fig. 2f). However, by plotting the number of evoked APs at each current intensity and fitting the data with a Boltzmann function, we found that the maximal number of spikes elicited from BTBR neurons dropped to ~50% of that from WT ones and the firing threshold for BTBR (0.2 nA) was higher than that for WT (0.1 nA) cells (Fig. 2g). This suggests that BTBR PNs are less excitable. To characterize the spike-frequency adaptation, we estimated the instantaneous spike frequencies at onset (*F*_O_) and in the steady state (*F*_SS_) toward the end of the pulses (Fig. 2e). As expected [38], both *F*_O_ and *F*_SS_ increased with greater depolarization while *F*_SS_ was smaller than *F*_O_ at any given current step (Supplementary Table S1 and Fig. 2h, i). Yet, BTBR PNs generated APs at a much lower *F*_O_, but not *F*_SS_, than WT ones, implying compromised initiation of repetitive firing. By measuring the first APs during a depolarization step to 1.1 nA as an example (Fig. 2j), we noticed that the decay time of BTBR APs increased (WT: 0.29 ± 0.01 ms, BTBR: 0.33 ± 0.01 ms, *F*_1,18_ = 4.623, *p* = 0.045) but their amplitude stayed the same as WT APs (WT: 95.1 ± 1.8 mV, BTBR: 93.4 ± 1.1 mV, *p* > 0.05). The slow repolarization may attribute to reduced expression/function of high-threshold K^+^ channels [39]. To analyze the non-linear dynamics of APs, we plotted their phase plane trajectories by displaying the change rate of membrane voltage (d*V*/d*t*) against the corresponding membrane potential (Fig. 2k, l). The spike threshold was defined as the potential where d*V*/d*t* reached 5% of its maximal value (d*V*/d*t* Max). While the threshold of BTBR APs moderately elevated (*F*_1,18_ = 4.177, *p* = 0.056), the d*V*/d*t* Max significantly decreased in BTBR as compared to WT group (*F*_1,18_ = 5.353, *p* = 0.033; Fig. 2m, n), suggesting a reduced availability of transient Na^+^ conductance [38]. Collectively, our results pinpoint two major cellular processes underlying the compromised activity of PNs in the BTBR brain: (a) over-inhibition by excessive AP-evoked GABA release from INs, and (b) hypo-excitability of PNs unable to generate spikes competently.

### Kv1.2 agonist rectifies presynaptic over-inhibition and alleviates sociable and emotional deficits of BTBR mice

**Fig. 3:**
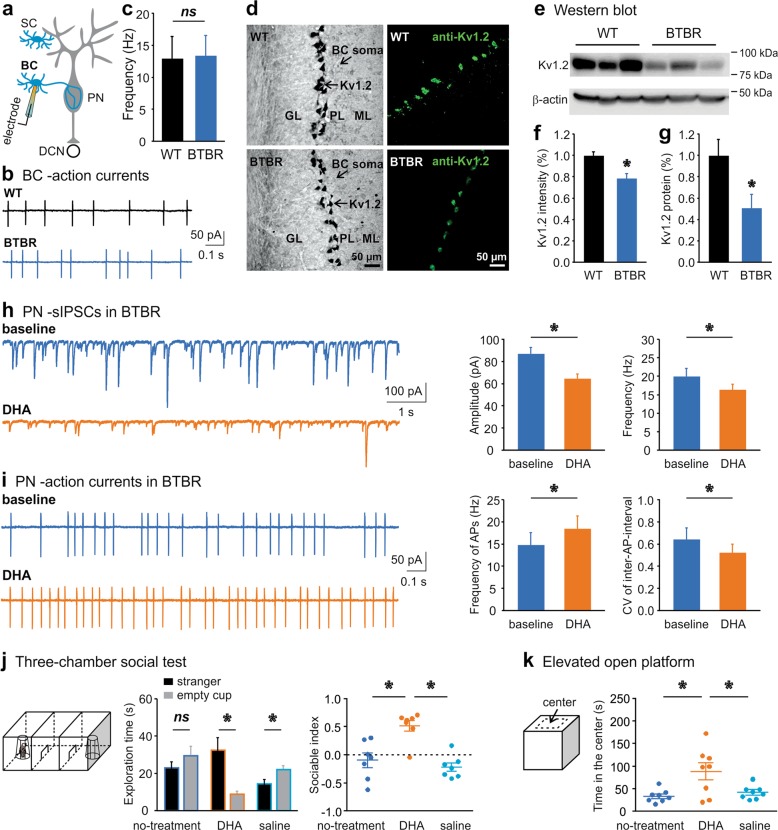
A Kv1.2 agonist alleviates inhibitory overtone in the cerebellar circuits and autistic behaviors of BTBR mice. **a** Schematics of recording configuration from a basket cell (BC) in NBQX (10 μM) and APV (50 μM) to isolate inhibitory inputs. **b** Cell-attached patch-clamp recordings of APs from a WT (top) and BTBR (bottom) BC soma. **c** Summary of firing frequency for WT (*n* = 8, black) and BTBR (*n* = 7, blue) BCs. **d** Confocal images of Kv1.2 immunolabeling (left: DAB staining; right: fluorescent staining) in WT (top) and BTBR (bottom) cerebellum. GL granular layer, PL Purkinje layer, ML molecular layer. **e** Western blot of Kv1.2 from WT and BTBR cerebellar homogenates. **f**, **g** Normalized (to the mean values of WT group) fluorescence intensity of Kv1.2 labeling (**f**) or amount of Kv1.2 protein (**g**) detected by Western blots for both groups (*n* = 3 mice for each). **h** sIPSCs (isolated by 10 µM NBQX and 50 μM APV) recorded from a PN in a BTBR brain slice before (baseline, blue) and after perfusion of DHA (100 µM, orange). Changes in amplitude and frequency of sIPSC are summarized on the right (*n* = 8). **i** APs elicited from a PN in the same condition as in **h**. DHA increases spike frequency (left) and decreases coefficient of variation (CV, right) of inter-AP-intervals of BTBR PNs (*n* = 7). **j** Illustration of three-chamber sociability test (trial 2) on the left. Time spent on exploring the stranger (black bars) or empty cup (gray bars) by BTBR mice (*n* = 8) is plotted for each condition: before and after DHA (200 mg/kg) or saline injection (middle). Compared to no- or saline-treatment, DHA increases sociable index (right). (**k**) Setup of elevated open platform, which is virtually divided into center and edge areas (left). Time spent in the center, an indicator of anxiety, is summarized for the three conditions (*n* = 8, right).

Among cerebellar INs, SCs innervate the distal dendrites of PNs, which locally counteract the excitation from PFs but do not directly affect spontaneous PN firing [40]. Conversely, BCs can robustly break down PN spiking by forming inhibitory synapses on the soma and ephaptic connections (pinceau) to the axon initial segment of PNs [41]. To test if increased activity of INs could account for the excessive GABA release at the BTBR synapses, we recorded APs from their soma while blocking excitatory inputs (Fig. 3a). Unexpectedly, there were no differences in firing frequencies between the WT and BTBR SCs (WT: 8.6 ± 1.3 Hz, BTBR: 9.2 ± 1.3 Hz; *p* > 0.05) or BCs (Fig. 3b, c), suggesting that the somatic excitability of INs is unaltered. Downstream mechanisms at their nerve terminals may be responsible for the unrestrained GABA release in the BTBR cerebellar cortex.

The membrane excitability of distinct compartments within a same neuron can be highly diverse due to non-uniform distribution of ion channels [39, 42]. In BCs, low-threshold K^+^ channels, Kv1.1 and Kv1.2, are concentrated in the axonal terminals and forcefully govern presynaptic excitability and vesicular fusion [43–47]. BC terminals contain the highest level of Kv1.2 in the entire brain [43, 44]. Kv1.2 is particularly important for axonal trafficking and surface expression of Kv1.1/Kv1.2 heteromers [48, 49]. In a FXS mouse model, we have shown that a reduction of Kv1.2 in the BC terminals elevates intracellular Ca^2+^ transients following AP invasions and induces more GABA release [31].

To determine if this is a common phenomenon in different ASD models, we labeled Kv1.2 by its antibody in the BTBR cerebellum. Figure 3d illustrated Kv1.2 staining clustered in the axonal terminal but not the soma of BCs as expected [43–46], which was confirmed by co-labeling with Calbindin (to mark PNs) and Parvalbumin (to mark PNs and INs) (Supplementary Fig. S2). To be consistent with the loci of electrophysiological recordings, we quantified Kv1.2 expression in the lobules V-VII and noticed a decrease of the Kv1.2 fluorescence intensity in the BTBR, as compared to the WT group (*F*_1,17_ = 36.533, *p* < 0.001; Fig. 3f). The overall number of BC terminals stained with the Kv1.2 antibody displayed no difference between the two groups (*F*_1,13_ = 0.280, *p* = 0.606). The results were corroborated by Western blots of cerebellar homogenates containing the vermis and hemisphere of the posterior cerebellum, which showed ~50% decline of Kv1.2 protein in the BTBR samples (*Z* = −2.169, *p* = 0.03; Fig. 3e, g).

Based on our data and the literature [31, 45, 49], we postulated that boosting the function of presynaptic Kv1.2 might revise the inhibitory overtone in the BTBR cerebellar circuitry. We selected a Kv1.2 agonist, DHA, which functions as a positive allosteric modulator of Kv1.2 channels by interacting with their voltage sensor to lower their activation threshold [31, 50, 51]. Perfusion of DHA to the BTBR brain slices effectively controlled the excessive GABA release, as manifested by the reduced amplitude (*F*_1,7_ = 30.194, *p* = 0.001) and frequency (*F*_1,7_ = 7.54, *p* = 0.029) of sIPSCs recorded from PNs (Fig. 3h). When examining the effect of DHA on the output neurons, we noted that DHA enhanced the firing frequency (*F*_1,6_ = 11.242, *p* = 0.015) and regularity (*F*_1,6_ = 6.453, *p* = 0.044) of BTBR PNs (Fig. 3i). Although DHA may not fully restore the PN activity to the WT level, presumably confined by their low intrinsic excitability (Fig. 1), the results demonstrate that DHA can alleviate the synaptic deficits by attenuating GABA release from INs in the BTBR cerebellum.

Subsequently, we assessed the impact of DHA on animal behaviors using a within-subject design with counterbalanced measures to minimize individual differences, i.e., same BTBR mice were rendered to behavioral testing before and after i.p. injection of DHA or saline. In the three-chamber sociability test (Fig. 3j), BTBR animals explored the stranger mouse and the empty cup indiscriminately, in line with early reports on their impaired social interaction [13, 15]. Following the DHA treatment, they spent more time exploring the stranger than the empty cup (*F*_1,6_ = 17.805, *p* = 0.006), reminiscent of normal behaviors of WT animals. In contrast, the saline injection did not correct the social avoidance of BTBR mice but rather worsened it (14.36 ± 1.84 s on “stranger” vs. 21.35 ± 1.95 s on “cup”, *F*_1,6_ = 7.363, *p* = 0.035). Further analysis of the sociable index revealed a significant “treatment” effect (*F*_2,12_ = 7.092, *p* = 0.009), meaning the index for the DHA treatment was much higher than that for the no-treatment (*p* = 0.048) or saline (*p* = 0.014) treatment. The total exploration time in each condition remained the same. In the social novelty trial, BTBR mice explored the novel stranger more than the previously encountered one, exhibiting intact social novelty preference as previously described [13, 15]. This was not affected by either DHA or saline administration (Supplementary Table S2).

To test if DHA could benefit psychiatric detriments of the ASD model, we chose the elevated open platform [15]. The anxiety level is inversely proportional to the length of time they stay in the central field (Fig. 3k). BTBR mice usually spend less time in the center than WT animals [15]. Interestingly, after DHA treatment, they were more present in the center, compared to the no-treatment (*p* = 0.02) or saline-injection (*p* = 0.048) conditions, giving a remarkable “treatment” effect when analyzed by the time spent in the central area (*F*_2,14_ = 7.064, *p* = 0.008), but not by the total traveling distance on the platform (Supplementary Table S2). Moreover, in an open-field test, there were no specific influences of DHA on locomotor activities of BTBR mice, as compared to the saline injection (*p* > 0.05; Supplementary Table S2). This is in agreement with our previous findings that DHA does not disturb general behaviors of WT mice including social interaction [31]. Thereby we suggest that downregulation of presynaptic Kv1.2 channels in the cerebellum is likely a conserved phenotype in ASD, and DHA rectifies the cellular and behavioral impairments of the BTBR strain. Despite of evident Kv1.2 congregation in the IN terminals [43, 44], the systemic rescues with DHA may have targeted other ion channels and other brain regions [51]. We favor DHA because of its safety (FDA-approved dietary supplement) and its ability to cross the blood-brain barrier [52], which presents a superb opportunity for translational applications.

### Chemogenetic excitation of PNs ameliorates ASD-like behaviors of BTBR mice

Given that many brain areas are remodeled in structure and function in BTBR mice, we wondered if normalizing PN activity alone would be sufficient to rescue their autistic phenotype. We employed a designer receptors exclusively activated by designer drugs (DREADD)-mediated chemogenetic approach by packaging a Pcp2/L7 mini-promoter with an hM3Dq receptor and a fluorescent protein (mCherry) in an AAV. The Pcp2 promoter is engineered to express proteins specifically in PNs [32]. Activation of hM3Dq by CNO is known to increase neuronal excitability [33].

**Fig. 4:**
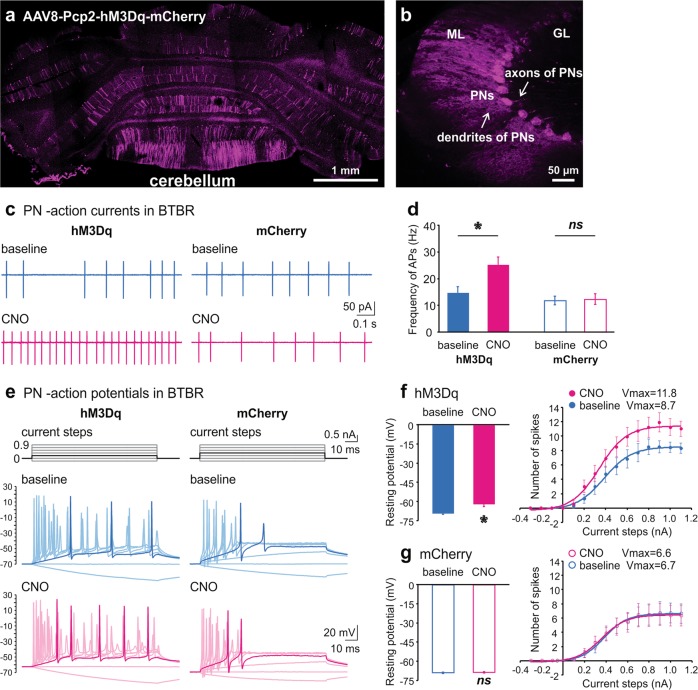
Expression of excitatory DREADD in PNs enhances their firing activity in the BTBR cerebellar cortex. **a** Example of AAV-carried DREADD expression in the BTBR cerebellum. **b** Selective targeting of PNs by AAV8-Pcp2-hM3Dq-mCherry (hM3Dq). ML molecular layer, GL granular layer. **c** APs elicited from PNs transduced with hM3Dq (left) or AAV8-Pcp2-mCherry (mCherry, right) before (blue) and after bath application of CNO (10 µM, magenta). **d** Frequencies of APs are quantified for the above conditions (*n* = 9 for hM3Dq, *n* = 6 for mCherry). **e** APs generated by depolarization steps (top) from a PN transduced with hM3Dq (left) or mCherry (right) before (blue) and after CNO perfusion (10 µM, magenta) in cocktail blockers of NBQX (10 μM), APV (50 μM) and bicuculline (10 μM). **f**, **g** Changes in membrane potentials (left) and numbers of spikes (right) made by CNO are summarized for hM3Dq (**f**, *n* = 10) and mCherry (**g**, *n* = 10) groups. Solid lines represent fits to a Boltzmann function: *f*(*I*) = Vmax/(1 + *e*^(Imid-I)/Ic^)+C, in which “Vmax” is theoretical value of the maximal number of APs, “Imid” is depolarization current needed to produce half of the maximal number of APs, and “Ic” is steepness of the Boltzmann curve.

First, we performed bilateral intracranial injection of the viral vector (AAV8-Pcp2-hM3Dq-mCherry) close to the neonatal cerebellum at P5. Four weeks later, we examined the brain-wide distribution of DREADD marked by mCherry and found the presence of DREADD only in the cerebellum. A coronal section revealed an efficient transduction of DREADD covering the entire cerebellar cortex (Fig. 4a). A higher magnification image demonstrated that DREADD was selectively expressed in the cell body, axon, and dendrites of PNs (Fig. 4b). We recorded APs from the transduced PNs of BTBR cerebellar slices in the cell-attached configuration without interfering synaptic transmission in the circuits. Activation of DREADD by CNO (10 µM) dramatically increased the firing frequency of PNs, overcoming the strong inhibition from INs in the BTBR synapses (*F*_1,8_ = 21.864, *p* = 0.002; Fig. 4c, d). The same treatment did not change the firing activity of PNs that were transduced by a control virus excluding DREADD (AAV8-Pcp2-mCherry, *p* > 0.05). To inspect the effect of CNO on the intrinsic excitability of PNs, we injected current steps to evoke spikes in the whole-cell mode after blocking all the synaptic inputs. Figure 4e exemplified that CNO depolarized the membrane potential, shortened onset of the first AP and increased the number of spikes in the hM3Dq-postive but not hM3Dq-negative neurons. Quantitatively, CNO raised the membrane potential of PNs expressing hM3Dq from −69.3 ± 0.74 to −62.2 ± 1.86 mV (*F*_1,8_ = 21.864, *p* = 0.002) without affecting the control ones (Fig. 4f, g, left). While CNO did not alter the input resistance of PNs measured by the steady-state potentials (as illustrated in Fig. 2f), it noticeably augmented the maximal number of spikes discharged from the hM3Dq-containing (*F*_1,9_ = 16.143, *p* = 0.003) but not hM3Dq-lacking (*F*_1,9_ = 1.499, *p* = 0.252) cells (Fig. 4f, g, right).

**Fig. 5:**
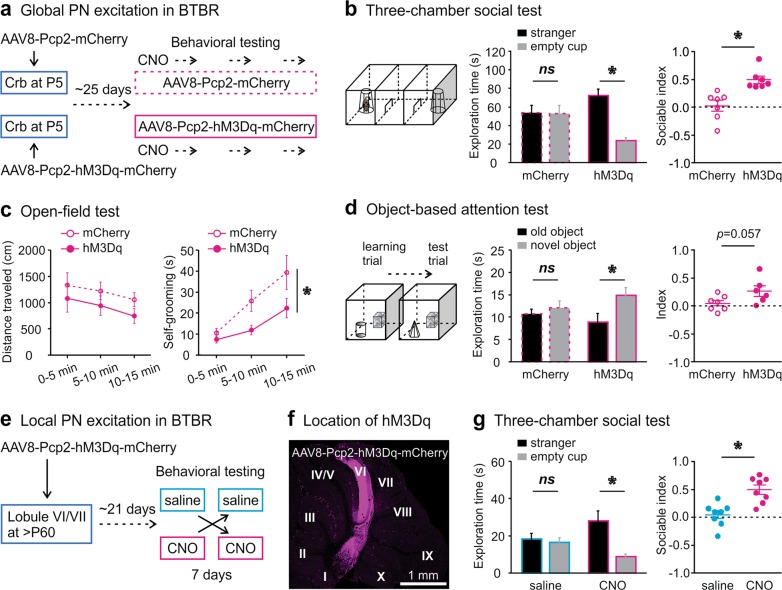
Behavioral rescues by chemogenetic excitation of PNs in the BTBR cerebellum. **a** Design for intracranial injection of AAV8-Pcp2-mCherry (mCherry) or AAV8-Pcp2-hM3Dq-mCherry (hM3Dq) at postnatal day (P) 5 to target PNs in the entire cerebellum (Crb) of BTBR mice as depicted in Fig. 4. After 25 days of transduction, animals are subjected to behavioral tests following CNO injections (1 mg/kg, i.p.). **b** In the sociability session of a three-chamber test (left), BTBR mice transduced with mCherry spent equal time exploring the stranger (black bars) and empty cup (gray bars) but BTBR mice transduced with hM3Dq approach the stranger more than empty cup (middle), rendering a higher sociable index (filled circles) than mCherry (empty circles) group (*n* = 7 for each, right). **c** Distance traveled (left) and time spent on grooming (right) in the open-field test are quantified for three successive periods. BTBR mice injected with hM3Dq (*n* = 7, filled circles) groom less than those with mCherry (*n* = 8, empty circles) in the last two intervals. **d** Schematics of an object-based attention test consisting of learning and test trials (left). In the test trial, hM3Dq group (*n* = 7) explore the new (gray bars) more than old (black bars) object while mCherry group (*n* = 8) do not (middle), generating a more positive index for hM3Dq (filled circles) than for mCherry (empty circles) cohort (right). **e**–**g** Within-subject design (**e**) for infusion of hM3Dq to target PNs in lobules VI & VIIa (**f**) of ~2-months-old BTBR mice via stereotaxic surgery. Three weeks later, animals are subjected to behavioral tests following alternating CNO (1 mg/kg) or saline administrations. In the three-chamber sociability test (**g**), animals treated by CNO (to activate hM3Dq receptors) explore the stranger more than empty cup, giving a greater sociable index (magenta) than the saline (blue) group (*n* = 8 for each).

Once the expression of excitatory DREADD and/or mCherry in PNs was established, we performed behavioral assays following i.p. injection of CNO in both groups (Fig. 5a). In the three-chamber sociability trial, significant effects of “object” (*F*_1,12_ = 15.134, *p* = 0.002) and “group × object” (*F*_1,12_ = 13.99, *p* = 0.003), but not “group” (*p* > 0.05), were found in the analysis of exploration time using a mixed two-way ANOVA. BTBR mice that received the control AAV explored the stranger and the empty cup equally (*p* > 0.05), similar to untreated ones [13, 15]. However, the hM3Dq group explored the stranger more than the empty cup (*t*_6_ = 5.728, *p* = 0.001), giving a higher sociable index than the control group (*F*_1,12_ = 16.296, *p* = 0.002; Fig. 5b). In the social novelty trial, the two cohorts did not indicate major differences in the overall exploration performance (Supplementary Table S3). This suggests that chemogenetic excitation of PNs rectifies the impaired social interaction, particularly social approaching, in BTBR mice. In the open field, we quantified travel distance and grooming behavior in three consecutive intervals by taking into account confounding factors (anxiety/habituation) that could influence rodent locomotor activity [53]. A significant effect of “interval” (*F*_2,24_ = 5.591, *p* = 0.01), but not “group” or “group × interval” (*p* > 0.05), was detected in the analysis of distance traveled, although subsequent one-way ANOVAs showed no group differences at any time period (*p* > 0.05; Fig. 5c). There were also no group differences in rearing behavior and time spent in the center (*p* > 0.05; Supplementary Table S3). By contrast, the assessments of time spent on grooming uncovered remarkable effects of “group” (*F*_1,13_ = 6.034, *p* = 0.029) and “interval” (*F*_2,26_ = 13.347, *p* < 0.001), but not their interaction (*p* > 0.05). Accordingly, the hM3Dq mice displayed less self-grooming than the control ones (*t*_13_ = −2.456, *p* = 0.029; Fig. 5c), indicating that restoring PN excitability alleviates repetitive stereotyped behavior of BTBR animals with a marginal weight on their gross movements. In the object-based attention test, no group differences were found in the total object-exploration time in either the learning or the test trial (*p* > 0.05; Supplementary Table S3). Yet, further scrutiny of the test trial revealed a significant “object” (*F*_1,11_ = 9.288, *p* = 0.011), but not “group” or “group × object” (*p* > 0.05), effect. Consistent with our earlier study [15], BTBR mice expressing only mCherry showed attention and/or memory deficits by exploring the old and new objects indiscernibly (*p* > 0.05), whereas the hM3Dq group preferred the novel to the old object (*t*_5_ = −2.731, *p* = 0.041) with an improved cognition index (*F*_1,11_ = 4.54, *p* = 0.057; Fig. 5d). The group difference became more obvious when the indexes were compared to the cutoff value (zero) (mCherry: *t*_6_ = 0.971, *p* = 0.369; hM3Dq: *t*_5_ = 2.844, *p* = 0.036), implying that reinstatement of PN firing compensates for the loss of cognitive functions in the BTBR strain.

Second, we infused AAV8-Pcp2-hM3Dq-mCherry into the posterior cerebellum (lobules VI & Crus 1) of adult BTBR mice by stereotaxic surgery (Fig. 5e), in light of clinical evidence on the anatomical basis for the cerebellum-associated pathogenesis of ASD [30, 54]. Three weeks after transduction, a robust yet restricted expression of DREADD appeared in the injected subregions (Fig. 5f). A within-subject design by testing the same BTBR mice transfected with the hM3Dq receptor after i.p. injection of CNO or saline was opted for behavioral evaluations (Fig. 5e). In the sociability trial of the three-chamber test, the animals explored the stranger more than empty cup when treated by CNO (*t*_7_ = 3.864, *p* = 0.006), but not by saline (*p* > 0.05), rendering a significant “treatment” effect on the social index (*F*_1,7_ = 15.378, *p* = 0.006; Fig. 5g). No group differences were observed in the total exploration time during the sociability trial or in the performance during the social novelty trial (*p* > 0.05; Supplementary Table S3). In the open field, there were no group differences in traveling distance, time in the center, rearing or self-grooming behaviors (*p* > 0.05; Supplementary Table S3). The same chemogenetic manipulation did not affect the behavioral readouts of WT animals either in the three-chamber or in the open-field tests (Supplementary Table S3), compatible with an earlier finding that increasing PN firing in adult mice has no impact on their sociability [55]. Our results suggest that restoring the activity of the posterior cerebellum is imperative for rescuing the social impairment of the BTBR model.

## Discussion

Different from the hippocampus where an increased ratio of excitation/inhibition is found in BTBR mice [56], the elevated inhibition from presynaptic INs together with lowered intrinsic excitability of postsynaptic PNs impedes the output activity of the BTBR cerebellar cortex. We have exploited a pharmacological approach to harness rampant GABA release from INs by enhancing the function of Kv1.2 channels, and a chemogenetic strategy to boost the membrane excitability of PNs. Both interventions effectively rectify the local circuitry dysfunctions and system-wide social, motor, affective and cognitive detriments of the idiopathic ASD model (Supplementary Fig. S3).

Despite being an established model for ASD, the genetic aspects of BTBR mice underlying their autistic-like behaviors are unclear. Multiple gene mutations with characteristic DNA polymorphisms in BTBR [17, 57] have made it difficult to map out the molecular basis for the aberrant presynaptic and postsynaptic excitability at the IN-PN synapses. Informed by an essential role of Kv1.2 in controlling the presynaptic excitability [43–47] and downregulation of Kv1.2 by deletion of *Fmr1* [31], we probed and found a reduced level of the K^+^ channels in the BTBR nerve terminals (Fig. 3d–g). Knowing that Fragile X Mental Retardation protein (FMRP) promotes the function of Kv1.2 in a phosphorylation-dependent manner [31], we quantified FMRP, phosphorylated-FMRP (p-FMRP), and other proteins in the mammalian target of rapamycin (mTOR) pathway, as well as metabotropic glutamate receptor 5 (mGluR5). Hyperactive mTOR and mGluR5 signaling are implicated in ASD and other psychiatric disorders [58, 59]. However, our analysis by Western blotting of cerebellar extracts from adult BTBR and WT mice disclosed no differences in these proteins (Supplementary Fig. S4). In the postsynaptic domain, low intrinsic excitability of PNs exacerbates the cellular phenotype of BTBR animals (Fig. 2e–n). Ion channels that are pivotal for PN membrane excitability include voltage-gated Na^+^ channels [60, 61], Ca^2+^-activated K^+^ channels [62], voltage-gated Ca^2+^ channels (VGCCs) [63], and HCN channels [64]. Of particular relevance to ASD, human genetic screenings highlight Nav1.1 (SCN1A), T-type VGCC (CACNA1H), and HCN [65–67]. Future investigations will determine the genome networks that constitutively modify the channels/proteins underpinning the aberrant neural excitability.

We utilized two delivery methods to elevate PN excitability with the chemogenetic approach at different spatial scales. Targeting PNs in the entire cerebellum relieved a wider spectrum of autistic symptoms in BTBR mice, comprising hyperactivity, attention and social deficits (Fig. 5). For the region-specific rescue, we focused on the posterior cerebellum containing the central vermis and lateral hemisphere, with the knowledge of its replicable association with ASD [30, 54]. Although both manipulations ameliorate social deficiency of BTBR mice, the particular benefits on the non-social phenotypes provided by recovering the whole cerebellum activity implicate essential involvements of other cerebellar subdivisions in ASD, for instance, the anterior cerebellum that mostly modulates the sensory-motor related cortices. Comprehensive examinations will clarify how the functional zones in the cerebellum contribute to distinct autistic features. In addition to the location difference, the global intervention was introduced at a younger age (Fig. 5). Its more robust outcomes may indicate a critical period for treating such a neurodevelopmental disorder. This is resonant of an elegant report on the sensitive periods for rescuing ASD-like behaviors in *Tsc1*-KO mice with an mTOR inhibitor rapamycin [68]. Future implementation of rescue paradigms with varying timing, dose and duration of DHA or chemogenetic administrations will help address the temporal constituent. Furthermore, application of DHA or CNO restored the cerebellar activity by affecting the rates and patterns of PN firing (Figs. 3, 4). Although the debate remains, a recent study meticulously shows the firing rates, but not the temporal patterns, of individual PNs dictate cerebellum-controlled movements [69]. Further inquiries are needed to answer whether a global change in the speed and/or a specific pattern of PN activity is crucial for the information processing in social behaviors.

It is surprising that restoring the cerebellar activity was sufficient to rescue the autistic behaviors of BTBR mice because these animals do not have corpus callosum, and the missing corpus callosum is implied as a cause for their autistic characters [70]. While incompletely penetrant, such a lesion is also observed in human ASD [71, 72]. But, contradicting evidence shows that postnatal ablation of the callosal inter-hemisphere connections in WT mice does not produce social or stereotypical phenotypes [73]. A recent study suggests that the inter-hemisphere connections in the posterior cerebellum of BTBR mice are abnormal, which may precipitate their behavioral deficits [74]. Therefore, the BTBR model may present as a unique and serendipitous tool for us to learn the complexity of the cerebellum-mediated alterations in ASD.

Although we have concentrated on the non-motor functions in BTBR mice, we recognize that the cerebellum engagements in the sensorimotor processing play a key role in development of ASD [75–78]. Interestingly, the abundance of Kv1.2 in the inhibitory cerebellar synapses is modulated by associative motor learning, such as eye-blink conditioning [79, 80], which is viewed as a biomarker for ASD [81]. Our findings on the common neuropathology, i.e., decreased expression of Kv1.2 at the IN terminals, among genetic (*Fmr1*-KO) and phenotypic (BTBR) autistic models rationalize Kv1.2 agonists, such as DHA, as a potential therapeutics for treating ASD. In fact, omega-3 fatty acids (including DHA) have been in clinical trials for ASD with conflicting results [82, 83], which reinforces the importance of understanding its complexity with various mouse lines. In the BTBR cerebellum, DHA promotes PN firing by limiting over-inhibition from INs. This might not be the case for other autistic models, for example, *Tsc1*-KO mice where reduced intrinsic excitability of PNs alone accounts for their behavioral phenotype [27]. An enhancement of K^+^ conductance by DHA would exacerbate the firing deficit of PNs and autistic behaviors in these mice.

Being the only output from the cerebellar cortex, low firing activity of PNs in nearly all ASD-like mouse strains [27–29, 31] may disinhibit downstream DCN, which sequentially gates the outgoing information to the thalamus and cortices, potentially influencing the integrative networks [84]. Utilizing the Pcp2 promoter-driven DREADD to “reset” the cerebro-cerebellar functional connectivity under region- and cell-type-specific control may provide an effective strategy for identification of potential therapies for ASD.

## Funding and disclosure

This work was supported by the National Institute of Neurological Disorders And Stroke (NINDS) of the National Institutes of Health (NIH) grant R15NS112964 to Y.M.Y. and the University of Minnesota faculty start-up fund to Y.M.Y. The authors declare no competing interests.

## Supplementary information

supplementary information
