# Supplementary material for: Targeting inhibitory cerebellar circuitry to alleviate behavioral deficits in a mouse model for studying idiopathic autism
Source: Neuropsychopharmacology. 2020 Mar 16;45(7):1159–70. doi: 10.1038/s41386-020-0656-5 (PMC7234983; doi:10.1038/s41386-020-0656-5)
Supplement: Supplementary file 1 — supplementary information [file 41386_2020_656_MOESM1_ESM.docx]

**Supplementary Materials and Methods**

***Subjects***

Black and Tan BRachyury T^+^Itpr3^tf^/J (BTBR, JAX stock #002282) and C57BL/6J (WT) mice were from Jackson Laboratory (Bar Harbor, ME) and housed in a facility accredited by the Association for the Assessment and Accreditation of Laboratory Animal Care (AAALAC). All mice were kept under a 12-hour light-dark cycle (light on from 07:00 to 19:00) and reared 3-5 per cage with food and water *ad libitum*. All procedures were approved by the Institutional Animal Care and Use Committee (IACUC) and the Institutional Biosafety Committee (IBC) of University of Minnesota, in accordance with the National Institutes of Health guidelines. Male mice were used unless otherwise specified. Choice of sexes was based on the male-dominant prevalence of ASD [1]. Different batches of animals were used for each set of experiments.

***Electrophysiology***

Mice at postnatal days (P) 28-35 were subject to electrophysiological (>3 mice/group) and other analyses (except for behavioral testing). Following decapitation with a small guillotine, the brain was immediately dissected and sagittal cerebellar slices were sectioned at a thickness of 300 µm using a vibratome (Leica Biosystems VT1200S, Buffalo Grove, IL) in ice-cold modified artificial cerebral spinal fluid (ACSF). It contained (in mM): sucrose (217.6), KCl (3), glucose (10), NaH_2_PO_4_ (2.5), NaHCO_3_ (26), MgCl_2_ (2), and CaCl_2_ (2), continuously bubbled in 95% O_2_ and 5% CO_2_ (pH 7.4). Subsequently, slices were incubated in oxygenated standard ACSF including (in mM): NaCl (125), KCl (2.5), glucose (10), NaH_2_PO_4_ (1.25), sodium pyruvate (2), myo-inositol (3), ascorbic acid (0.5), NaHCO_3_ (26), MgCl_2_ (1), and CaCl_2_ (2) (pH 7.4) at 37°C for 30 min prior to experimentation.

Slices were transferred to a recording chamber mounted on an Olympus microscope with a 60X water immersion objective. They were continuously perfused with the standard ACSF at a rate of ~1 ml/min with supplement of NBQX (10 µM) and APV (50 µM) to block AMPA and NMDA receptors or bicuculline (10 µM) to block GABA_A_ receptors. Purkinje neurons (PNs) and interneurons (INs) were identified by their size and location. Patch electrodes had resistances of 2.5-3 and 4.5-6 MΩ for PNs and INs, respectively. Spontaneous and miniature excitatory postsynaptic currents (EPSCs) and inhibitory postsynaptic currents (IPSCs) from PNs were recorded in the whole-cell voltage-clamp mode at a holding potential of -60 mV without liquid junction correction. Tetrodotoxin (TTX, 1 µM), a Na^+^ channel blocker, was added to isolate miniature EPSCs and IPSCs by inhibiting action potentials (APs). To record EPSCs, the intracellular solution contained (in mM): K-gluconate (97.5), CsCl (32.5), EGTA (5), HEPES (10), MgCl_2_ (1), TEA (30), and lidocaine *N-*ethyl bromide (3) (pH 7.3). To record IPSCs, the intracellular solution contained (in mM): K-gluconate (50), CsCl (80), EGTA (5), HEPES (10), MgCl_2_ (1), TEA (30) and lidocaine N-ethyl bromide (3) (pH 7.3). Spontaneous APs at the soma were recorded in the cell-attached mode with GΩ seal at -60 mV for PNs and -70 mV for INs, which registered as compound inward-outward currents. In this case, the intracellular solution included (in mM): K-gluconate (97.5), KCl (32.5), EGTA (0.1), HEPES (40), MgCl_2_ (1), ATP (2), GTP (0.5) (pH 7.3). The same solution was used to record spikes from PNs in the current-clamp mode at a holding potential of -70 mV while synaptic inputs were eliminated by NBQX (10 µM), APV (50 µM) and bicuculline (10 µM). For analyses of spikes evoked by current-step injections, curve fittings were done with Clampfit.

All recordings were acquired on-line at room temperature (~23°C), filtered at 4 kHz, digitized at 50 kHz with a dual-channel amplifier (Molecular Devices MultiClamp 700B, San Jose, CA) and digitizer (Molecular Devices Digidata 1550B). Recordings not reaching initial GΩ seal or holding currents higher than 300 pA were omitted. Data were analyzed off-line with MiniAnalysis 6.0.7 (Synaptosoft, Fort Lee, NJ), Clampfit 10 (Molecular Devices) and Excel 2016 (Microsoft, Redmond, WA). Reagents were from Millipore Sigma (St. Louis, MO), Tocris Bioscience (Bristol, UK) and Alomone Labs (Jerusalem, Israel).

***Immunohistochemistry***

Mice (3-4 mice/group) were deeply anesthetized, and perfused with ice-cold phosphate-buffered saline (PBS, pH 7.4), followed by 4% paraformaldehyde. Brains were extracted, immersed in 4% paraformaldehyde at 4°C overnight, and transferred to 30% sucrose solution at 4°C until they sank. They were sectioned into 50 µm-thick slices on a microtome (Leica Biosystems SM2010R, Buffalo Grove, IL), maintained in 0.3% H_2_O_2_ solution for 10 min, and rinsed in PBS. Brain slices were permeabilized with 0.2% Triton X-100 in 2% goat serum blocking solution for 30 min at 37°C, and labelled with primary antibodies diluted in the same blocking solution for 48 h at 4°C. Slices were then washed with 0.2% Triton X-100 solution and incubated in biotinylated anti-mouse or anti-rabbit antibody (1:200; Vector Laboratories BA-9200, Burlingame, CA) for 2 h at room temperature. After being rinsed in 0.2% Triton X-100, slices were incubated in ABC reagent (Vector Laboratories PK-4000) for 1 h. Following a rinse by PBS, slices were stained with 3,3’-diaminobenzidine (DAB substrate kit; Vector Laboratories SK-4100) for 3-5 min, then washed with distilled water, mounted and cover-slipped (VectaMount Permanent; Vector Laboratories H-5000). To add fluorescence, after incubation in primary antibodies, slices were maintained for 1 h at 37°C, rinsed by PBS, incubated in secondary antibodies (1:1000; Invitrogen Alex Fluor 488, 555 or 647, Waltham, MA) for 3 h at room temperature, and then mounted and cover-slipped with mounting medium (Invitrogen P36966).

Primary antibodies included: anti-Kv1.2 (1:1000; Sigma-Aldrich SAB5200059), anti-Calbindin-D28 (1:1000; Invitrogen PA1-931), and anti-Parvalbumin (1:1000; Novus Biologicals NBP2-50036).

***Western blotting***

The cerebellum of BTBR and WT mice (3 mice/group) were dissected and snap-frozen in dry ice. Brain tissues were homogenized in ice-cold lysis buffer (20 mM HEPES pH7.5, 100 mM NaCl, 0.05% Triton X-100, 1 mM DTT, 5 mM Na-Betaglycerophosphate, 0.5 mM Na-[vanadate](https://en.wikipedia.org/wiki/Sodium_vanadate), 1 mM EDTA, protease inhibitors) and protein was extracted and quantified by Bradford assay. 50 µg protein lysates were resolved on a 10% SDS polyacrylamide gel and transferred to PVDF membrane (Bio-Rad, Hercules CA). Membranes were blocked by 5% non-fat dry milk and incubated with primary antibodies overnight at 4°C. Next day blots were washed and incubated in horseradish peroxidase (HRP) conjugated with anti-rabbit or anti-mouse IgG antibodies (1:5000; Bio-Rad) for 1.5 h and washed again. To detect immunoreactivity, blots were incubated with enhanced chemiluminescent reagent (Perkin Elmer, Waltham, MA) and imaged on Odyssey Fc Imaging System (LI-COR Biosciences, Lincoln, NE). Relative intensity of blots was quantified using ImageJ-NIH software.

Primary antibodies were: anti-Kv1.2 (1:1000; Sigma-Aldrich SAB5200059), anti-FMRP (1:100; NovusBio NBP2-01770, Littleton, CO), anti-pFMRP (1:50; Abcam ab48127, Cambridge, MA), anti-mTOR (1:1000; Cell Signaling 2983, Danvers, MA), anti-p-mTOR (1:1000; Cell Signaling 2971), anti-ERK (1:1000; Santa Cruz sc-514302, Dallas, TX), anti-p-ERK (1:1000; Cell Signaling 9101), anti-mGluR5 (1:100; Invitrogen PA1-38132), and anti-β-actin (1:5000; Sigma-Aldrich A5441).

***DHA administration in vivo***

2-3 months old BTBR mice (n=8) underwent behavioral testing before and after intraperitoneal (i.p.) injection of cis-4,7,10,13,16,19-Docosahexaenoic acid sodium salt (DHA; Millipore Sigma D8768) or sterile saline. Each condition was separated by an inter-treatment interval of 7 days. Tests were conducted 30 min after DHA (200 mg/kg) or saline injection to minimize distress from drug administration and ensure normal physical activity that could be affected by DHA for a short period [2].

***AAV-vector delivery***

AAV8-Pcp2-hM3Dq-mCherry and AAV8-Pcp2-mCherry vectors (titers > 4.0×10^13^) were prepared by the University of Minnesota Viral Vector and Cloning Core following standard packaging procedures. Minimal Pcp2/L7 promoter was inserted into pAAV-CaMKIIa-hM3D(Gq)-mCherry (a gift from Dr. Bryan Roth; Addgene plasmid #50476, Watertown, MA) to confer specific expression of human M3 muscarinic receptor (hM3Dq) and/or mCherry to PNs [3]. Activation of hM3Dq by clozapine-*N*-oxide (CNO, Enzo BML-NS105, Farmingdale, NY) increased neuronal excitability [4].

*Intracranial injection to cover the whole cerebellum*

BTBR pups at P5 were randomly assigned to AAV8-Pcp2-hM3Dq-mCherry or AAV8-Pcp2-mCherry group (n=8 mice/group) and anesthetized by hypothermia. The viral vectors (1:3 dilution) were slowly injected with a 10 µl Hamilton syringe into the loci close to the cerebellum bilaterally (1.5-2.0 µl each side). Pups were then kept on a heating pad (~ 37°C) until fully recovered and placed back to their nursing mother. At ~P30, the animals underwent a battery of behavioral tests following CNO administration (1 mg/kg, i.p.). The low dose of CNO intended to minimize hM3Dq-independent effects.

*Infusion into the cerebellum subregions via stereotaxic surgery*

2-3 months old BTBR (6 males and 3 females) or WT (7 males) mice were anaesthetized with ketamine (100 mg/kg, i.p.) and xylazine (10 mg/kg, i.p.). Subcutaneous injection of meloxicam (5 mg/kg) was used as an analgesic. Mice were mounted on a stereotaxic frame (Stoelting, Wood Dale, IL) and holes were drilled according to the coordinates: AP: -7.0 mm, ML: ±1.0 mm, relative to the bregma, to target lobules VI and VIIa. AAV8-Pcp2-hM3Dq-mCherry (0.2 µl each side) was infused with a 33-gauge steel cannula inserted 0.5 mm below brain surface and connected to a 10 µl Hamilton syringe. A microinjection pump (CMA 100, Holliston, MA) controlled the flowrate at 0.1 µl/min. After infusion, the needle remained in place for 7 min and was slowly retracted to minimize viral backflow. The wound was sutured and animals were monitored until fully recovered. 3 weeks after surgery, behavioral tests were conducted with a within-group design: half of the mice were injected with CNO (1 mg/kg, i.p.) and the other with saline; 1 week later the previous CNO group received saline, and vice versa.

***Behavioral testing***

Apparatus and procedures were previously described in detail [5]. Behaviors were recorded via a camera connected to a tracking software ANY-maze (Stoelting). Experimenters were blind to the treatments. DHA, CNO or saline was given 30-40 min before each test.

*Three-chamber social test*

The test was to evaluate mouse sociable behaviors, which consisted of three trials [6]. In the habituation trial, the subject was placed into the middle chamber and allowed to freely explore all three chambers. In the sociability trial, a sex- and age-matched stranger mouse that had never contacted the subject was put underneath a grid cup located in a side-chamber. Another identical empty cup was placed on the other side. The locations for placing the stranger and empty cup were counterbalanced between subjects. In the social novelty trial, another stranger was put inside the previously empty cup. The subject was allowed to explore the “familiar” and the “novel” strangers. Each trial lasted for 9 min. Physical contacts around the cups with the nose, head and forelimbs were defined as exploration behaviors. To minimize individual differences, sociable index = (time for exploring the stranger - time for exploring the empty cup) / (total exploration time); and social novelty index = (time for exploring the novel stranger - time for exploring the familiar stranger) / (total exploration time) were calculated. Positive values represented intact sociability and social novelty preference.

*Elevated open platform*

The test exploited the innate tendency of fear of open space in rodents; and less staying time in the center is a sign of anxiety [5]. Animals were put onto an open platform that was elevated 30 cm and allowed to freely explore for 5 min. Distance travelled and time spent in the center area were recorded.

*Open field*

The test was to measure general locomotor and exploratory activities [7]. The animal was put into the arena to freely behave for 10-15 min. Distance travelled, rearing, grooming, thigmotactic behaviors, entries and time stayed at the center (virtual central square 13.3 × 13.3 cm) were analyzed.

*Object-based attention test*

The test was to measure attention-associated processes and/or working (short-term) memory [5]. It was identical to a novel object-preference test, except with no time delay between learning and test trials. In the learning trial, two distinct objects were placed in the arena and the animal explored them freely. In the test trial, both objects were replaced by a novel object and an object identical to either explored ones. Each trial lasted for 5 min. Object exploration was defined by physical contacts. An index was calculated: index = (time for exploring the novel object - time for exploring the old object) / (total object exploration time). Positive values indicated intact object-based attention and/or short-term memory.

***Statistics***

Mixed two-way ANOVAs with a “between-subject” factor (group) and a “within-subject” factor (condition, object or interval), and repeated two-way ANOVAs with “within-subject” factors (treatment, condition, object and interval) were applied to analyze the data. When a significant effect was detected, one-way or repeated one-way ANOVAs and LSD *post hoc* tests were conducted. Independently, paired Student's *t*-tests and Mann-Whitney *U*-test were used when appropriate. One-sample *t*-tests were applied to compare the indexes to zero value. All statistics were two-tailed and significant levels were set as *p*<0.05. Details were summarized in **Supplementary Table S1**. Data were expressed as the mean ± standard error (s.e.m.). For behavioral testing, *n* represented the number of mice. For other analyses, *n* denoted the number of cells or samples from >3 animals in each group. Sample sizes were decided by previous studies using similar experimental protocols [2,5].

**References**

1 Baio J, Wiggins L, Christensen DL, Maenner MJ, Daniels J, Warren Z, et al. Prevalence of Autism Spectrum Disorder Among Children Aged 8 Years - Autism and Developmental Disabilities Monitoring Network, 11 Sites, United States, 2014. MMWR Surveill Summ. 2018;67(6):1-23.

2 Yang Y-M, Arsenault J, Bah A, Krzeminski M, Fekete A, Chao OY, et al. Identification of a molecular locus for normalizing dysregulated GABA release from interneurons in the Fragile X brain. Molecular Psychiatry. 2018.

3 Nitta K, Matsuzaki Y, Konno A, Hirai H. Minimal Purkinje Cell-Specific PCP2/L7 Promoter Virally Available for Rodents and Non-human Primates. Mol Ther Methods Clin Dev. 2017;6:159-70.

4 Roth BL. DREADDs for Neuroscientists. Neuron. 2016;89(4):683-94.

5 Chao OY, Yunger R, Yang YM. Behavioral assessments of BTBR T+Itpr3tf/J mice by tests of object attention and elevated open platform: Implications for an animal model of psychiatric comorbidity in autism. Behav Brain Res. 2018;347:140-47.

6 Silverman JL, Yang M, Lord C, Crawley JN. Behavioural phenotyping assays for mouse models of autism. Nat Rev Neurosci. 2010;11(7):490-502.

7 Prut L, Belzung C. The open field as a paradigm to measure the effects of drugs on anxiety-like behaviors: a review. Eur J Pharmacol. 2003;463(1-3):3-33.

**
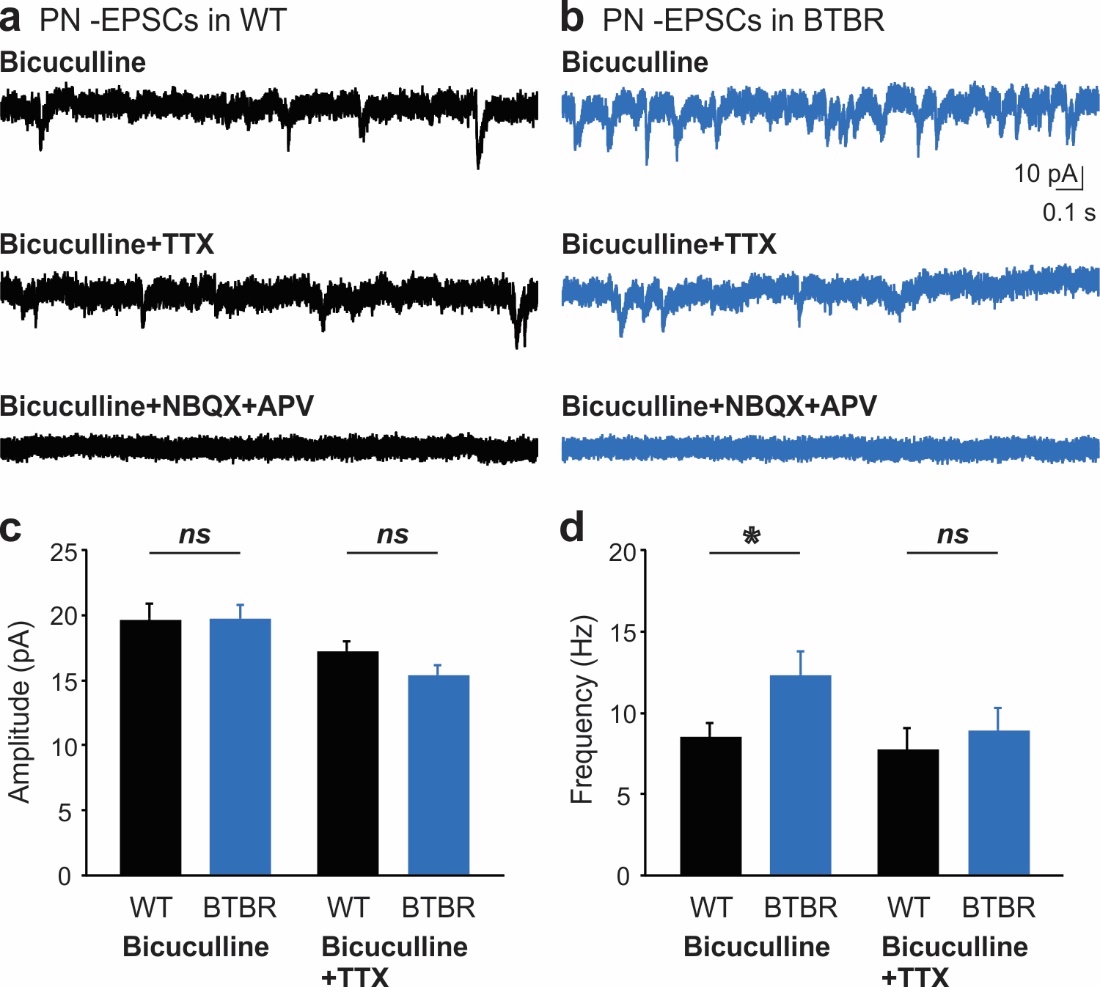
**

**Supplementary Fig. S1 Glutamatergic inputs to PNs in the BTBR synapses.** (**a**, **b**) EPSCs recorded in the whole-cell mode at a holding potential of -60 mV from PNs of WT (**a**) and BTBR (**b**) mice in the presence of bicuculline (10 μM) to block inhibitory inputs. The same PNs are then exposed to TTX (1 μM) or a combination of NBQX (10 μM) and APV (50 μM). (**c**, **d**) Summary of the amplitude (**c**) and frequency (**d**) of EPSCs for WT (black bars) and BTBR (blue bars) PNs before (n=11 for WT, n=12 for BTBR) and after TTX exposure (n=12 for WT, n=9 for BTBR). The frequency is calculated as the reciprocal of each inter-EPSC-intervals and averaged for each neuron.

**
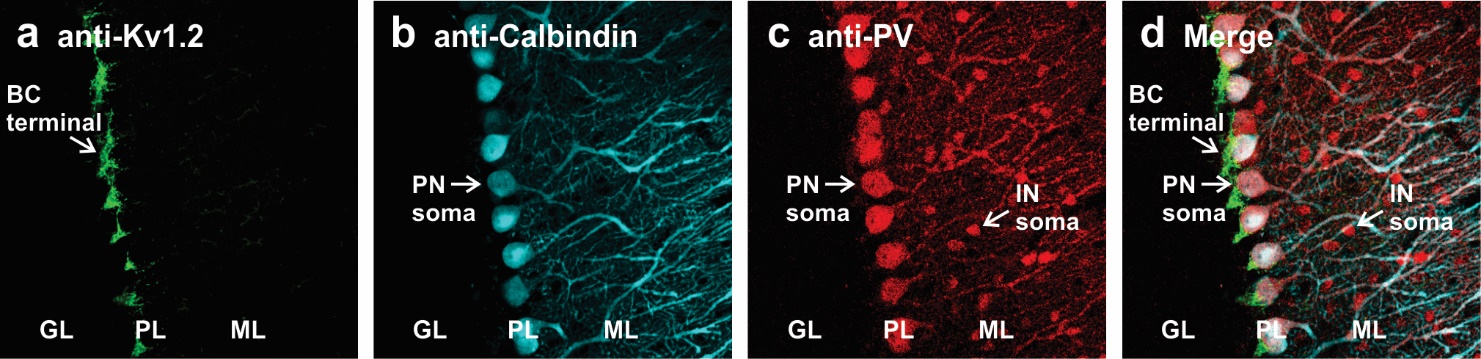
**

**Supplementary Fig. S2 Co-labelling of Kv1.2, Calbindin and Parvalbumin (PV).** (**a**-**d**) Confocal images of fluorescent immunostaining with antibodies against Kv1.2 (green), Calbindin (cyan), PV (red) and their mergence in a cerebellar slice from a WT mouse. Kv1.2 clusters in the BC axonal terminals surrounding PNs, which are labelled with anti-Calbindin antibody. Anti-PV antibody stains the soma of PNs and INs (including BCs). GL: granular layer, PL: Purkinje layer, ML: molecular layer.

**
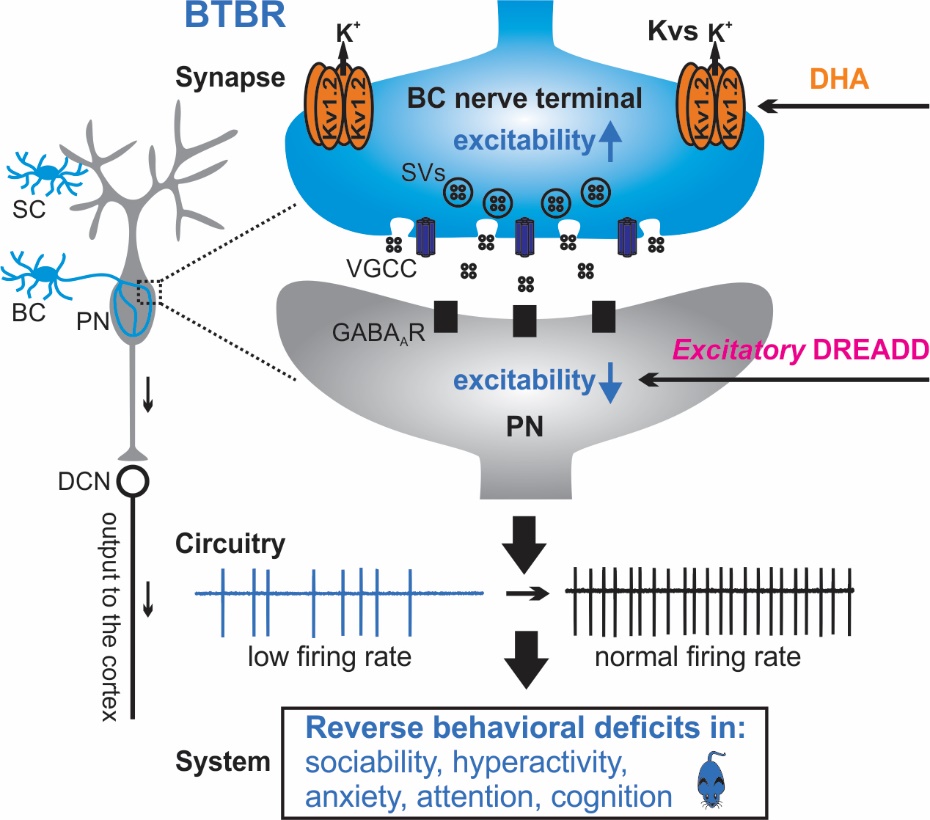
**

**Supplementary Fig. S3 A graphical model for targeting the cerebellar circuitry in the ASD mouse line.** In the BTBR cerebellum, loss of Kv1.2 at the IN nerve terminals elevates presynaptic excitability and Ca^2+^ inflow through voltage-gated calcium channels (VGCC), leading to excessive GABA release. Concurrent impairment in the intrinsic excitability of postsynaptic PNs further dampens the output activity from the cerebellar cortex. Kv1.2 agonist (DHA) and specific promoter-guided DREADD restore the presynaptic and postsynaptic excitability respectively in the inhibitory circuitry, providing novel regimes for system rescue of ASD-like behaviors.

**
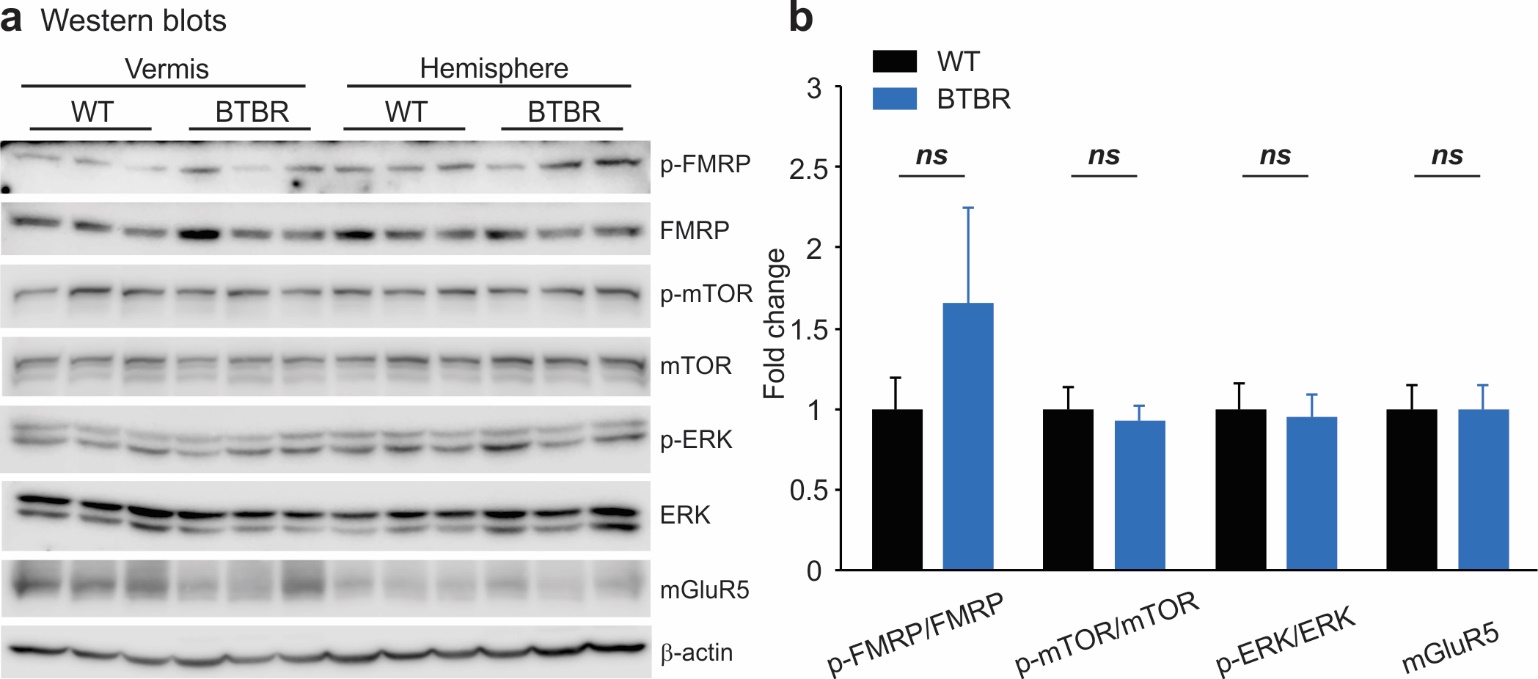
**

**Supplementary Fig. S4 Proteins related to mTOR and mGluR5 signaling in the BTBR cerebellum.** (**a**) Western blots of Fragile X Mental Retardation protein (FMRP), phosphorylated (p)-FMRP, mammalian target of rapamycin (mTOR), p-mTOR, extracellular signal-regulated kinase (ERK), p-ERK, and metabotropic glutamate receptor 5 (mGluR5). β-actin is used to normalize amount of loaded proteins, extracted from central vermis or lateral hemisphere of the posterior cerebellum in adult WT or BTBR mice. (**b**) Fold changes of proteins for the two groups (n=3 mice for each group, n=6 samples for each protein by pooling vermis and hemisphere) are quantified by normalizing to the average values of WT mice. Ratios of phosphorylated to non-phosphorylated proteins are calculated.

**Supplementary Table S1. List of statistics applied for data analyses.**

| **Experiments** | **Methods** | **Results** |
| --- | --- | --- |
| Compare APs of PNs in WT and BTBR | Mixed two-way ANOVA | “group” effect F1,18=5.633, p=0.029  “condition” effect F2,36=36.235, p<0.001  “group×condition” effect F2,36=1.648, p=0.207 |
|  | One-way ANOVAs for each condition | control:  “group” effect F1,18=4.679, p=0.044  NBQX+APV:  “group” effect F1,18=4.719, p=0.043  NBQX+APV+bicuculline:  “group” effect F1,18=6.171, p=0.023 |
|  | Repeated one-way ANOVA within each group | WT:  “condition” effect F2,18=23.842, p<0.001  BTBR:  “condition” effect F2,18=12.726, p<0.001 |
|  | Post-hoc LSD within each group | WT:  control vs. NBQX+APV, p=0.305  control vs. NBQX+APV+bicuculline, p=0.001  NBQX+APV vs. NBQX+APV+bicuculline, p<0.001  BTBR:  control vs. NBQXAPV, p=0.606  control vs. NBQX+APV+bicuculline, p=0.009  NBQX+APV vs. NBQX+APV+bicuculline, p=0.003 |
| Compare CV of PN firing in WT and BTBR | Mixed two-way ANOVA | “group” effect F1,18=10.876, p=0.004  “condition” effect F2,36=8.184, p=0.001  “group×condition” effect F2,36=2.733, p=0.079 |
|  | One-way ANOVAs for each condition | control:  “group” effect F1,18=9.115, p=0.007  NBQX+APV:  “group” effect F1,18=6.764, p=0.018  NBQX+APV+bicuculline:  “group” effect F1,18=7.745, p=0.012 |
|  | Repeated one-way ANOVA within each group | WT:  “condition” effect F2,18=20.855, p<0.001  BTBR:  “condition” effect F2,18=5.164, p=0.017 |
|  | Post-hoc LSD within each group | WT:  control vs. NBQX+APV, p=0.039  control vs. NBQX+APV+bicuculline, p=0.001  NBQX+APV vs. NBQX+APV+bicuculline, p=0.002  BTBR:  control vs. NBQX+APV, p=0.998  control vs. NBQX+APV+bicuculline, p=0.027  NBQX+APV vs. NBQX+APV+bicuculline, p=0.060 |
| Compare PN-IPSC of WT and BTBR | One-way ANOVAs | sIPSCs amplitude:  “group” effect F1,30=16.75, p<0.001  sIPSCs frequency:  “group” effect F1,30=4.926, p=0.034  mIPSCs amplitude:  “group” effect F1,20=0.042, p=0.84  mIPSCs frequency:  “group” effect F1,20=2.052, p=0.167 |
| Compare PN-EPSC of WT and BTBR | One-way ANOVAs | sEPSCs amplitude:  “group” effect F1,21=0.002, p=0.967  sEPSCs frequency:  “group” effect F1,21=5.544, p=0.028  mEPSCs amplitude:  “group” effect F1,19=3.497, p=0.077  mEPSCs frequency:  “group” effect F1,19=0.402, p=0.534 |
| Compare PN intrinsic excitability in WT and BTBR | One-way ANOVAs | Vmax:  “group” effect F1,18=6.926, p=0.017  Imid:  “group” effect F1,18=3.797, p=0.067  Ic:  “group” effect F1,18=0.101, p=0.754  C:  “group” effect F1,18=2.546, p=0.128 |
| Compare APs of PNs in WT and BTBR at different current steps | One-way ANOVAs | 0.5 nA F_O_:  “group” effect F1,17=5.153, p=0.037  0.5 nA F_SS_:  “group” effect F1,17=0.361, p=0.556  0.7 nA F_O_:  “group” effect F1,18=6.959, p=0.017  0.7 nA F_SS_:  “group” effect F1,18=0.525, p=0.478  0.9 nA F_O_:  “group” effect F1,18=7.964, p=0.011  0.9 nA F_SS_:  “group” effect F1,18=0.063, p=0.804   - 1. nA F_O_:   “group” effect F1,18=10.105, p=0.005   - 1. nA F_SS_:   “group” effect F1,18=0.819, p=0.377 |
| Compare 1^st^ APs of PNs at a current step of 1.1 nA in WT and BTBR | One-way ANOVAs | amplitude (mV):  “group” effect F1,18=0.662, p=0.426  halfwidth (ms):  “group” effect F1,18=8.781, p=0.008   - 1. rise time (ms):   “group” effect F1,18=3.382, p=0.082  90-10 decay time (ms):  “group” effect F1,18=4.623, p=0.045  dV/dt Max:  “group” effect F1,18=5.353, p=0.033  Threshold (at 5% of dV/dt Max):  “group” effect F1,18=4.177, p=0.056 |
| Compare APs of BCs in WT and BTBR | One-way ANOVA | “group” effect F1,13=0.009, p=0.926 |
| Compare APs of SCs in WT and BTBR | One-way ANOVA | “group” effect F1,12=0.106, p=0.751 |
| Compare density and number of Kv1.2 in WT and BTBR | One-way ANOVAs | fluorescence density:  “group” effect F1,17=36.533, p<0.001  Number of terminals stained by Kv1.2:  “group” effect F1,13=0.280, p=0.606 |
| Compare protein levels of WT and BTBR | Independent t-tests | Kv1.2: t10=2.380, p=0.039  mGluR5: t10=0.076, p=0.941  pFMRP/FMRP: t10=-1, p=0.341  pmTOR/TOR: t10=0.299, p=0.771  pERK/ERK: t10=0.243, p=0.813 |
| DHA effect on PN-APs in BTBR | Repeated one-way ANOVAs | frequency:  “DHA” effect F1,6=11.242, p=0.015  CV:  “DHA” effect F1,6=6.453, p=0.044 |
| DHA effect on PN-IPSCs in BTBR | Repeated one-way ANOVAs | amplitude:  “DHA” effect F1,7=30.194, p=0.001  frequency:  “DHA” effect F1,7=7.54, p=0.029 |
| DREADD effect on BTBR PNs | Repeated one-way ANOVAs | hM3Dq-mCherry:  APs “CNO” effect F1,8=21.864, p=0.002  Resting MP “CNO” effect F1,9=17.682, p=0.002  Vmax: “CNO” effect F1,9=16.143, p=0.003  Imid: “CNO” effect F1,9=1.871, p=0.205  Ic: “CNO” effect F1,9=0.717, p=0.419  C: “CNO” effect F1,9=7.491, p=0.023  mCherry:  APs “CNO” effect F1,5=0.492, p=0.514  Resting MP “CNO” effect F1,9=0.061, p=0.811  Vmax: “CNO” effect F1,9=1.499, p=0.252  Imid: “CNO” effect F1,9=0.019, p=0.894  Ic: “CNO” effect F1,9=0.011, p=0.917  C: “CNO” effect F1,9=3.109, p=0.112 |
| **DHA behavior in BTBR:** |  |  |
| Three-chamber sociability trial | Repeated two-way ANOVAs | “treatment” effect F2,12=3.122, p=0.081  “object” effect F1,6=1.072, p=0.34  “treatment×object” effect F2,12=9.343, p=0.004 |
|  | Repeated one-way ANOVAs within each treatment | No-treatment:  “object” effect F1,6=0.783, p=0.41  DHA:  “object” effect F1,6=17.805, p=0.006  saline:  “object” effect F1,6=7.363, p=0.035 |
|  | Paired t-tests within treatment | DHA:  stranger vs. cup, t6=4.22, p=0.006  saline:  stranger vs. cup, t6=-2.713, p=0.035 |
|  | Repeated one-way ANOVA for index | “treatment” effect F2,12=7.092, p=0.009 |
|  | Post-hoc LSD for index | No-treatment vs. DHA, p=0.048  No-treatment vs. saline, p=0.441  DHA vs. saline, p=0.014 |
|  | One-sample t-tests for index comparing to 0 | No-treatment: t6=-0.756, p=0.479  DHA, t6=6.807, p<0.001  saline, t6=2.859, p=0.029 |
|  | Repeated one-way ANOVA for total exploration | “treatment” effect F2,12=3.122, p=0.081 |
| Three-chamber social novelty trial | Repeated two-way ANOVAs | “treatment” effect F2,12=6.79, p=0.011  “object” effect F1,6=17.538, p=0.006  “treatment×object” effect F2,12=6.729, p=0.011 |
|  | Repeated one-way ANOVAs within each treatment | No-treatment:  “object” effect F1,6=11.667, p=0.014  DHA:  “object” effect F1,6=7.947, p=0.03  saline:  “object” effect F1,6=7.545, p=0.033 |
|  | Paired t-tests within treatment | No-treatment:  old vs. novel, t6=-3.416, p=0.014  DHA:  old vs. novel, t6=-2.819, p=0.03  saline:  old vs. novel, t6=-2.747, p=0.033 |
|  | Repeated one-way ANOVA for index | “treatment” effect F2,12=3.866, p=0.051 |
|  | One-sample t-tests for index compared to 0 | No-treatment: t6=3.517, p=0.013  DHA, t6=-2.871, p=0.028  saline, t6=3.504, p=0.013 |
|  | Repeated one-way ANOVA for total exploration | “treatment” effect F2,12=6.790, p=0.011 |
|  | Post-hoc LSD for total exploration | No-treatment vs. DHA, p=0.057  No-treatment vs. saline, p=0.018  DHA vs. saline, p=0.393 |
| Elevated open platform | Repeated one-way ANOVAs | distance:  “treatment” effect F2,14=2.061, p=0.164  center staying:  “treatment” effect F2,14=7.064, p=0.008 |
|  | Post-hoc LSD for center staying | No-treatment vs. DHA, p=0.02  No-treatment vs. saline, p=0.149  DHA vs. saline, p=0.048 |
| Open field | Repeated two-way ANOVAs | distance:  “treatment” effect F2,14=22.035, p<0.001  “interval” effect F1,7=26.859, p=0.001  “treatment×interval” effect F2,14=2.397, p=0.127  rearing:  “treatment” effect F2,14=11.899, p=0.001  “interval” effect F1,7=6.99, p=0.033  “treatment×interval” effect F2,14=6.637, p=0.009  grooming:  “treatment” effect F2,14=3.527, p=0.057  “interval” effect F1,7=2.339, p=0.17  “treatment×interval” effect F2,14=1.41, p=0.277  center entry:  “treatment” effect F2,14=9.983, p=0.002  “interval” effect F1,7=6.448, p=0.039  “treatment×interval” effect F2,14=0.564, p=0.581  center staying:  “treatment” effect F2,14=2.725, p=0.1  “interval” effect F1,7=2.762, p=0.14  “treatment×interval” effect F2,14=0.026, p=0.974  thigmotaxis:  “treatment” effect F2,14=13.05, p=0.001  “interval” effect F1,7=21.091, p=0.003  “treatment×interval” effect F2,14=1.262, p=0.313 |
|  | Post-hoc LSD for treatments | distance:  No-treatment vs. DHA, p=0.002  No-treatment vs. saline, p=0.002  DHA vs. saline, p=0.074  rearing:  No-treatment vs. DHA, p=0.093  No-treatment vs. saline, p=0.002  DHA vs. saline, p=0.017  center entry:  No-treatment vs. DHA, p=0.014  No-treatment vs. saline, p=0.011  DHA vs. saline, p=0.108  thigmotaxis:  No-treatment vs. DHA, p=0.001  No-treatment vs. saline, p=0.013  DHA vs. saline, p=0.356 |
| **DREADD global behavior in BTBR:** |  |  |
| Three-chamber sociability trial | Mixed two-way ANOVA | “group” effect F1,12=0.555, p=0.47  “object” effect F1,12=15.134, p=0.002  “group×object” effect F1,12=13.99, p=0.003 |
|  | Paired t-tests within each group | control:  stranger vs. cup, t6=-0.101, p=0.923  DREADD:  stranger vs. cup, t6=5.728, p=0.001 |
|  | One-way ANOVA for index | “group” effect F1,12=16.296, p=0.002 |
|  | One-sample t-tests for index compared to 0 | control: t6=0.301, p=0.774  DREADD: t6=7.479, p<0.001 |
|  | One-way ANOVA for total exploration | “group” effect F1,12=0.555, p=0.47 |
| Three-chamber social novelty trial | Mixed two-way ANOVA | “group” effect F1,12=0.002, p=0.965  “object” effect F1,12=6.372, p=0.027  “group×object” effect F1,12=1.083, p=0.319 |
|  | Paired t-tests within each group | control:  familiar vs. novel, t6=-3.353, p=0.015  DREADD:  familiar vs. novel, t6=-2.085, p=0.082 |
|  | One-way ANOVA for index | “group” effect F1,12=0.673, p=0.428 |
|  | One-sample t-tests for index compared to 0 | control: t6=3.219, p=0.018  DREADD: t6=2.150, p=0.075 |
|  | One-way ANOVA for total exploration | “group” effect F1,12=0.002, p=0.965 |
| Open field | Mixed two-way ANOVAs | distance:  “group” effect F1,12=1.239, p=0.287  “interval” effect F2,24=5.591, p=0.01  “group×interval” effect F2,24=0.055, p=0.946  rearing:  “group” effect F1,12=0.019, p=0.892  “interval” effect F2,24=1.528, p=0.237  “group×interval” effect F2,24=2.494, p=0.104  grooming:  “group” effect F1,13=6.034, p=0.029  “interval” effect F2,26=13.347, p<0.001  “group×interval” effect F2,26=1.503, p=0.241  center entry:  “group” effect F1,12=0.471, p=0.505  “interval” effect F2,24=0.184, p=0.833  “group×interval” effect F2,24=0.635, p=0.538  center staying:  “group” effect F1,12=0.295, p=0.597  “interval” effect F2,24=1.257, p=0.303  “group×interval” effect F2,24=0.248, p=0.782  thigmotaxis:  “group” effect F1,12=0.032, p=0.862  “interval” effect F2,24=15.177, p<0.001  “group×interval” effect F2,24=0.895, p=0.422 |
|  | One-way ANOVAs for each interval | distance_interval 1:  “group” effect F1,12=0.513, p=0.488  distance_interval 2:  “group” effect F1,12=1.24, p=0.287  distance_interval 3:  “group” effect F1,12=2.569, p=0.135  thigmotaxis_interval 1:  “group” effect F1,12=0.485, p=0.5  thigmotaxis _interval 2:  “group” effect F1,12=0.117, p=0.739  thigmotaxis _interval 3:  “group” effect F1,12=0.217, p=0.65 |
| Object-based attention (learning trial) | Paired t-tests within each group | control  object1 vs. object 2, t6=-0.258, p=0.805  DREADD  Object 1 vs. object 2, t5=-0.109, p=0.918 |
| Object-based attention (testing trial) | Mixed two-way ANOVA | “group” effect F1,11=0.07, p=0.796  “object” effect F1,11=9.288, p=0.011  “group×object” effect F1,11=3.514, p=0.088 |
|  | Paired t-tests within each group | control  old vs. novel, t6=-1.137, p=0.299  DREADD  old vs. novel, t5=-2.731, p=0.041 |
|  | One-way ANOVA for index | “group” effect F1,11=4.54, p=0.057 |
|  | One-sample t-tests for index compared to 0 | Control: t6=0.971, p=0.369  DREADD: t5=2.844, p=0.036 |
|  | One-way ANOVA for total exploration | “group” effect F1,11=1.805, p=0.206 |
| **DREADD local behavior in BTBR:** |  |  |
| Three-chamber sociability trial | Repeated two-way ANOVA | “treatment” effect F1,7=0.314, p=0.592  “object” effect F1,7=14.567, p=0.007  “treatment×object” effect F1,7=11.309, p=0.012 |
|  | Paired t-tests within each treatment | saline:  stranger vs. cup, t7=0.868, p=0.414  CNO:  stranger vs. cup, t7=3.864, p=0.006 |
|  | Repeated one-way ANOVAs | total exploration:  “treatment” effect F1,7=0.314, p=0.592  Index:  “treatment” effect F1,7=15.387, p=0.006 |
|  | One sample t-tests for index comparing to 0 | saline: t7=0.667, p=0.526  CNO: t7=6.165, p<0.001 |
| Three-chamber social novelty trial | Repeated two-way ANOVA | “treatment” effect F1,7=0.235, p=0.643  “object” effect F1,7=15.961, p=0.005  “treatment×object” effect F1,7=4.995, p=0.061 |
|  | Paired t-tests within each treatment | saline:  stranger vs. cup, t7=-3.861, p=0.006  CNO:  stranger vs. cup, t7=-3.231, p=0.014 |
|  | Repeated one-way ANOVAs | total exploration:  “treatment” effect F1,7=0.235, p=0.643  Index:  “treatment” effect F1,7=1.564, p=0.251 |
|  | One sample t-tests for index comparing to 0 | saline: t7=5.972, p=0.001  CNO: t7=3.996, p=0.005 |
| Open field | Repeated two-way ANOVAs | distance:  “treatment” F1,7=0.001, p=0.981  “interval” F2,14=99.793, p<0.001  “treatment×interval” F2,14=0.418, p=0.666  rearing:  “treatment” F1,7=0.723, p=0.423  “interval” F2,14=6.527, p=0.01  “treatment×interval” F2,14=0.145, p=0.867  grooming:  “treatment” F1,7=0.141, p=0.719  “interval” F2,14=7.25, p=0.007  “treatment×interval” F2,14=0.359, p=0.705  center entry:  “treatment” F1,7=1.445, p=0.268  “interval” F2,14=5.726, p=0.015  “treatment×interval” F2,14=1.922, p=0.183  center staying:  “treatment” F1,7=0.944, p=0.364  “interval” F2,14=1.289, p=0.306  “treatment×interval” F2,14=2.099, p=0.159  thigmotaxis:  “treatment” F1,7=0.147, p=0.713  “interval” F2,14=66.627, p<0.001  “treatment×interval” F2,14=0.867, p=0.442 |
|  | Repeated one-way ANOVAs | distance_interval 1:  “treatment” effect F1,7=0.108, p=0.752  distance_interval 2:  “treatment” effect F1,7=0.014, p=0.909  distance_interval 3:  “treatment” effect F1,7=1.296, p=0.292  rearing_interval 1:  “treatment” effect F1,7=0.172, p=0.691  rearing_interval 2:  “treatment” effect F1,7=0.183, p=0.681  rearing_interval 3:  “treatment” effect F1,7=4.667, p=0.068  grooming_interval 1:  “treatment” effect F1,7=2.045, p=0.196  grooming_interval 2:  “treatment” effect F1,7=0.061, p=0.812  grooming_interval 3:  “treatment” effect F1,7=0.264, p=0.623  center entry_interval 1:  “treatment” effect F1,7=0.2, p=0.668  center entry_interval 2:  “treatment” effect F1,7=1.944, p=0.206  center entry_interval 3:  “treatment” effect F1,7=1.895, p=0.211  thigmotaxis_interval 1:  “treatment” effect F1,7=0.461, p=0.519  thigmotaxis _interval 2:  “treatment” effect F1,7=0.203, p=0.666  thigmotaxis _interval 3:  “treatment” effect F1,7=3.428, p=0.107 |
| **DREADD local behavior in WT:** |  |  |
| Three-chamber sociability trial | Repeated two-way ANOVA | “treatment” effect F1,6=1.399, p=0.282  “object” effect F1,6=12.6, p=0.012  “treatment×object” effect F1,6=2.698, p=0.152 |
|  | Paired t-tests within each treatment | saline:  stranger vs. cup, t6=3.397, p=0.015  CNO:  stranger vs. cup, t6=3.061, p=0.022 |
|  | Repeated one-way ANOVAs | total exploration:  “treatment” effect F1,6=1.399, p=0.282  Index:  “treatment” effect F1,6=0.982, p=0.36 |
|  | One sample t-tests for index comparing to 0 | saline: t6=4.391, p=0.005  CNO: t6=3.35, p=0.015 |
| Three-chamber social novelty trial | Repeated two-way ANOVA | “treatment” effect F1,6=0.486, p=0.512  “object” effect F1,6=15.783, p=0.007  “treatment×object” effect F1,6=1.404, p=0.281 |
|  | Paired t-tests within each treatment | saline:  stranger vs. cup, t6=2.225, p=0.068  CNO:  stranger vs. cup, t6=4.762, p=0.003 |
|  | Repeated one-way ANOVAs | total exploration:  “treatment” effect F1,6=0.486, p=0.512  Index:  “treatment” effect F1,6=3.409, p=0.114 |
|  | One sample t-tests for index comparing to 0 | saline: t6=2.49, p=0.047  CNO: t6=4.261, p=0.005 |
| Open field | Repeated two-way ANOVAs | distance:  “treatment” F1,6=0.013, p=0.912  “interval” F2,12=4.667, p=0.032  “treatment×interval” F2,12=1.177, p=0.341  rearing:  “treatment” F1,6=0.081, p=0.785  “interval” F2,12=0.323, p=0.73  “treatment×interval” F2,12=1.29, p=0.311  grooming:  “treatment” F1,6=0.653, p=0.45  “interval” F2,12=3.286, p=0.73  “treatment×interval” F2,12=0.339, p=0.719  center entry:  “treatment” F1,6=1.04, p=0.347  “interval” F2,12=3.376, p=0.69  “treatment×interval” F2,12=2.515, p=0.122  center staying:  “treatment” F1,6=1.845, p=0.223  “interval” F2,12=1.948, p=0.185  “treatment×interval” F2,12=3.509, p=0.646  thigmotaxis:  “treatment” F1,6=0.004, p=0.951  “interval” F2,12=8.825, p=0.004  “treatment×interval” F2,12=1.167, p=0.344 |
|  | Repeated one-way ANOVAs | thigmotaxis_interval 1:  “treatment” effect F1,6=0.102, p=0.76  thigmotaxis _interval 2:  “treatment” effect F1,6=0.235, p=0.645  thigmotaxis _interval 3:  “treatment” effect F1,6=1.476, p=0.27 |

**Supplementary Table S2. Behavioral assessments in DHA experiments.** One animal was excluded as it did not explore the presented stimuli in the three-chamber social test (3CH). EOP = elevated open platform. OFT = open-field test. †p<0.05, compared to cup exploration; *p<0.05, compared to no-treatment; #p<0.05, compared to saline. Values represent mean ± s.e.m.

|  |  |  |  |  |  |  |  |  |  |  |  |  |  |  |  |
| --- | --- | --- | --- | --- | --- | --- | --- | --- | --- | --- | --- | --- | --- | --- | --- |
|  |  | **No-treatment** | |  |  |  | **DHA** |  |  |  |  | **saline** |  |  |  |
| **3CH** |  | n=8-1 |  |  |  |  | n=8-1 |  |  |  |  | n=8-1 |  |  |  |
| *trial2* |  | *stranger* |  | *cup* |  |  | *stranger* |  | *cup* |  |  | *stranger* |  | *cup* |  |
|  | sec. | 23.00 | ±3.13 | 29.63 | ±4.88 |  | 32.63 | ±6.48† | 8.93 | ±1.39 |  | 14.44 | ±2.12† | 22.07 | ±2.10 |
| total | sec. | 52.63 | ±3.34 |  |  |  | 41.56 | ±7.50 |  |  |  | 36.51 | ±3.14 |  |  |
| index |  | -0.10 | ±0.13 |  |  |  | 0.45 | ±0.16*# |  |  |  | -0.22 | ±0.08 |  |  |
| *trial3* |  | *familiar* |  | *novel* |  |  | *familiar* |  | *novel* |  |  | *familiar* |  | *novel* |  |
|  | sec. | 10.56 | ±1.90† | 39.81 | ±8.68 |  | 10.13 | ±1.60† | 17.19 | ±2.49 |  | 8.26 | ±1.63† | 14.63 | ±2.51 |
| total | sec. | 50.37 | ±9.19 |  |  |  | 27.31 | ±3.35 |  |  |  | 22.89 | ±3.54 |  |  |
| index |  | 0.53 | ±0.08 |  |  |  | 0.25 | ±0.07 |  |  |  | 0.26 | ±0.08 |  |  |
|  |  |  |  |  |  |  |  |  |  |  |  |  |  |  |  |
| **EOP** |  | n=8 |  |  |  |  | n=8 |  |  |  |  | n=8 |  |  |  |
| distance | cm | 1936.98 | ±188.43 |  |  |  | 1659.01 | ±151.74 |  |  |  | 1566.86 | ±145.13 |  |  |
| center | sec. | 33.65 | ±4.90 |  |  |  | 88.40 | ±18.56*# |  |  |  | 41.68 | ±5.27 |  |  |
|  |  |  |  |  |  |  |  |  |  |  |  |  |  |  |  |
| **OFT** |  | n=8 |  |  |  |  | n=8 |  |  |  |  | n=8 |  |  |  |
|  | min | *0-5* |  | *5-10* |  |  | *0-5* |  | *5-10* |  |  | *0-5* |  | *5-10* |  |
| distance | cm | 2517.14 | ±229.44 | 1438.29 | ±136.02 |  | 1921.91 | ±222.92 | 586.65 | ±109.63* |  | 1463.55 | ±251.24 | 689.98 | ±120.92* |
| rearing | counts | 12.75 | ±3.06 | 9.75 | ±1.91 |  | 15.50 | ±5.03 | 2.25 | ±0.80# |  | 8.63 | ±3.29 | 2.38 | ±0.80* |
| grooming | sec. | 17.74 | ±4.70 | 48.53 | ±15.22 |  | 37.01 | ±6.72 | 66.33 | ±12.91 |  | 66.04 | ±12.05 | 56.10 | ±15.30 |
| center | counts | 7.88 | ±1.41 | 5.50 | ±0.98 |  | 5.50 | ±0.73 | 2.38 | ±0.46* |  | 3.63 | ±0.91 | 2.00 | ±0.65* |
| center | sec. | 4.14 | ±0.80 | 3.39 | ±0.67 |  | 2.96 | ±0.68 | 2.09 | ±0.74 |  | 2.21 | ±0.79 | 1.59 | ±0.75 |
| thigmotaxis | cm | 1755.11 | ±212.67 | 876.00 | ±104.46 |  | 1119.09 | ±184.71 | 277.95 | ±50.34* |  | 1103.39 | ±181.11 | 523.08 | ±88.00* |
|  |  |  |  |  |  |  |  |  |  |  |  |  |  |  |  |

**Supplementary Table S3. Behavioral assessments in DREADD experiments.** One or two animals were excluded as they did not explore the presented stimuli in the three-chamber social test (3CH) and object-based attention test (OBAT) or were immobile over 8 min in the open field test (OFT). †p<0.05, compared to cup or novel object exploration; *p<0.05, compared to the respective control. Values represent mean ± s.e.m.

|  |  |  |  |  |  |  |  |  |  |  |  |  |  |  |
| --- | --- | --- | --- | --- | --- | --- | --- | --- | --- | --- | --- | --- | --- | --- |
| ***Global DREADD in BTBR*** |  | **control_AAV8-Pcp2-mCherry** | | | |  |  |  | **DREADD_AAV8-Pcp2-hM3Dq-mCherry** | | | | |  |
| **3CH** |  | n=8-1 |  |  |  |  |  |  | n=8-1 |  |  |  |  |  |
| *trial2* |  | *stranger* |  | *cup* |  |  |  |  | *stranger* |  | *cup* |  |  |  |
|  | sec. | 53.93 | ±7.68 | 52.97 | ±8.47 |  |  |  | 72.44 | ±6.72† | 23.74 | ±3.00 |  |  |
| total | sec. | 106.9 | ±13.06 |  |  |  |  |  | 96.19 | ±6.00 |  |  |  |  |
| index |  | 0.03 | ±0.09 |  |  |  |  |  | 0.49 | ±0.07* |  |  |  |  |
| *trial3* |  | *familiar* |  | *novel* |  |  |  |  | *familiar* |  | *novel* |  |  |  |
|  | sec. | 21.13 | ±5.66† | 44.06 | ±12.04 |  |  |  | 28.21 | ±4.57 | 37.76 | ±6.13 |  |  |
| total | sec. | 65.19 | ±14.92 |  |  |  |  |  | 65.97 | ±9.10 |  |  |  |  |
| index |  | 0.29 | ±0.15 |  |  |  |  |  | 0.14 | ±0.10 |  |  |  |  |
|  |  |  |  |  |  |  |  |  |  |  |  |  |  |  |
| **OFT** |  | n=8 |  |  |  |  |  |  | n=8-1 |  |  |  |  |  |
|  | min | *0-5* |  | *5-10 min* |  | *10-15 min* | |  | *0-5 min* |  | *5-10 min* |  | *10-15 min* | |
| distance | cm | 1334.44 | ±235.56 | 1221.90 | ±165.05 | 1060.26 | ±131.36 |  | 1081.50 | ±259.90 | 944.47 | ±185.11 | 746.78 | ±142.33 |
| rearing | counts | 2.00 | ±1.31 | 4.00 | ±1.84 | 5.00 | ±1.40 |  | 4.71 | ±2.64 | 5.57 | ±3.85 | 4.00 | ±1.99 |
| grooming | sec. | 10.60 | ±2.21 | 25.91 | ±5.03 | 39.46 | ±8.11 |  | 7.49 | ±1.64 | 11.94 | ±2.26 | 22.34 | ±4.67* |
| center | counts | 6.25 | ±1.92 | 7.38 | ±2.17 | 9.13 | ±2.55 |  | 6.43 | ±1.90 | 6.57 | ±2.03 | 6.00 | ±2.93 |
| center | sec. | 14.96 | ±7.59 | 8.21 | ±3.70 | 7.88 | ±2.18 |  | 19.57 | ±13.72 | 14.64 | ±10.56 | 7.07 | ±4.73 |
| thigmotaxis | cm | 913.28 | ±154.69 | 718.24 | ±81.46 | 532.03 | ±49.50 |  | 1072.22 | ±163.77 | 668.12 | ±130.94 | 492.02 | ±74.38 |
|  |  |  |  |  |  |  |  |  |  |  |  |  |  |  |
| **OBAT** |  | n=8 |  |  |  |  |  |  | n=8-1 for learning and n=8-2 for testing | | | | |  |
| *learning trial* | | *object1* |  | *object2* |  |  |  |  | *object1* |  | *object2* |  |  |  |
|  |  | 9.28 | ±1.85 | 10.28 | ±1.87 |  |  |  | 14.04 | ±4.01 | 15.21 | ±2.06 |  |  |
| total |  | 19.55 | ±2.61 |  |  |  |  |  | 29.26 | ±5.58 |  |  |  |  |
| *test trial* |  | *old* |  | *novel* |  |  |  |  | *old* |  | *novel* |  |  |  |
|  |  | 10.74 | ±1.01 | 12.16 | ±1.49 |  |  |  | 8.95 | ±1.85† | 14.88 | ±1.68 |  |  |
|  |  | 22.9 | ±2.22 |  |  |  |  |  | 23.83 | ±2.79 |  |  |  |  |
|  |  | 0.05 | ±0.05 |  |  |  |  |  | 0.27 | ±0.09 |  |  |  |  |

| ***Local***  ***DREADD***  ***in BTBR*** | | **saline_AAV8-Pcp2-hM3Dq-mCherry** | | | |  |  |  | **CNO_AAV8-Pcp2-hM3Dq-mCherry** | | | |  |  |
| --- | --- | --- | --- | --- | --- | --- | --- | --- | --- | --- | --- | --- | --- | --- |
| **3CH** |  | n=8 |  |  |  |  |  |  | n=8 |  |  |  |  |  |
| *trial2* |  | *stranger* |  | *cup* |  |  |  |  | *stranger* |  | *cup* |  |  |  |
|  | sec. | 18.34 | ±3.00 | 16.64 | ±2.38 |  |  |  | 28.03 | ±5.33† | 8.83 | ±1.41 |  |  |
| total | sec. | 34.98 | ±5.05 |  |  |  |  |  | 36.85 | ±6.02 |  |  |  |  |
| index |  | 0.05 | ±0.07 |  |  |  |  |  | 0.50 | ±0.08* |  |  |  |  |
| *trial3* |  | *familiar* |  | *novel* |  |  |  |  | *familiar* |  | *novel* |  |  |  |
|  | sec. | 9.44 | ±1.66 | 28.29 | ±5.59 |  |  |  | 12.04 | ±3.39 | 22.30 | ±3.32 |  |  |
| total | sec. | 37.73 | ±6.65 |  |  |  |  |  | 34.34 | ±5.90 |  |  |  |  |
| index |  | 0.47 | ±0.08 |  |  |  |  |  | 0.36 | ±0.09 |  |  |  |  |
|  |  |  |  |  |  |  |  |  |  |  |  |  |  |  |
| **OFT** |  | n=8 |  |  |  |  |  |  | n=8 |  |  |  |  |  |
|  | min | *0-5 min* |  | *5-10min* |  | *10-15 min* | |  | *0-5 min* |  | *5-10min* |  | *10-15 min* | |
| distance | cm | 2160.49 | ±252.52 | 1059.61 | ±172.23 | 769.56 | ±211.82 |  | 2039.69 | ±186.01 | 1039.83 | ±131.53 | 896.30 | ±166.26 |
| rearing | counts | 6.88 | ±1.83 | 5.00 | ±1.27 | 1.88 | ±0.83 |  | 7.75 | ±2.37 | 6.13 | ±2.17 | 3.88 | ±1.33 |
| grooming | sec. | 4.89 | ±0.85 | 16.51 | ±3.42 | 37.64 | ±10.64 |  | 6.75 | ±1.20 | 15.69 | ±3.89 | 31.01 | ±12.28 |
| center | counts | 6.63 | ±0.86 | 3.50 | ±1.02 | 3.00 | ±0.85 |  | 7.13 | ±1.27 | 6.00 | ±1.81 | 4.38 | ±1.50 |
| center | sec. | 7.20 | ±3.19 | 3.43 | ±0.75 | 1.60 | ±0.46 |  | 4.88 | ±0.95 | 8.43 | ±3.35 | 5.15 | ±2.76 |
| thigmotaxis | cm | 1605.16 | ±230.07 | 749.38 | ±104.32 | 513.11 | ±127.21 |  | 1421.28 | ±114.88 | 694.73 | ±95.73 | 611.08 | ±121.63 |
|  |  |  |  |  |  |  |  |  |  |  |  |  |  |  |
| ***Local***  ***DREADD***  ***in WT*** | | **saline_AAV8-Pcp2-hM3Dq-mCherry** | | | |  |  |  | **CNO_AAV8-Pcp2-hM3Dq-mCherry** | | | |  |  |
| **3CH** |  | n=7 |  |  |  |  |  |  | n=7 |  |  |  |  |  |
| *trial2* |  | *stranger* |  | *cup* |  |  |  |  | *stranger* |  | *cup* |  |  |  |
|  | sec. | 94.41 | ±14.25† | 39.14 | ±6.81 |  |  |  | 73.81 | ±10.93† | 38.30 | ±6.31 |  |  |
| total | sec. | 133.56 | ±15.31 |  |  |  |  |  | 112.11 | ±13.57 |  |  |  |  |
| index |  | 0.39 | ±0.09 |  |  |  |  |  | 0.32 | ±0.09 |  |  |  |  |
| *trial3* |  | *familiar* |  | *novel* |  |  |  |  | *familiar* |  | *novel* |  |  |  |
|  | sec. | 49.69 | ±7.70 | 71.09 | ±6.81 |  |  |  | 39.63 | ±9.25 | 72.50 | ±8.81 |  |  |
| total | sec. | 120.77 | ±10.91 |  |  |  |  |  | 112.13 | ±16.69 |  |  |  |  |
| index |  | 0.19 | ±0.08 |  |  |  |  |  | 0.33 | ±0.08 |  |  |  |  |
|  |  |  |  |  |  |  |  |  |  |  |  |  |  |  |
| **OFT** |  | n=7 |  |  |  |  |  |  | n=7 |  |  |  |  |  |
|  | min | *0-5 min* |  | *5-10min* |  | *10-15 min* | |  | *0-5 min* |  | *5-10min* |  | *10-15 min* | |
| distance | cm | 899.13 | ±183.88 | 586.11 | ±88.09 | 633.00 | ±56.39 |  | 912.87 | ±132.87 | 671.70 | ±65.72 | 563.69 | ±18.41 |
| rearing | counts | 10.57 | ±3.52 | 6.57 | ±1.53 | 8.43 | ±0.78 |  | 8.86 | ±1.88 | 9.43 | ±1.38 | 8.29 | ±1.02 |
| grooming | sec. | 6.97 | ±3.21 | 14.63 | ±5.59 | 20.06 | ±10.41 |  | 3.16 | ±0.71 | 12.57 | ±4.28 | 11.40 | ±4.14 |
| center | counts | 1.86 | ±0.67 | 2.86 | ±0.83 | 3.29 | ±0.81 |  | 2.43 | ±0.69 | 5.00 | ±1.00 | 2.14 | ±0.77 |
| center | sec. | 2.27 | ±1.07 | 5.51 | ±2.06 | 6.20 | ±2.73 |  | 1.77 | ±0.53 | 4.10 | ±0.91 | 3.70 | ±1.83 |
| thigmotaxis | cm | 669.06 | ±131.00 | 372.43 | ±56.83 | 409.16 | ±38.66 |  | 703.81 | ±99.98 | 412.24 | ±43.82 | 348.31 | ±22.87 |
|  |  |  |  |  |  |  |  |  |  |  |  |  |  |  |
